# The structure of a GH149 β‐(1 → 3) glucan phosphorylase reveals a new surface oligosaccharide binding site and additional domains that are absent in the disaccharide‐specific GH94 glucose‐β‐(1 → 3)‐glucose (laminaribiose) phosphorylase

**DOI:** 10.1002/prot.25745

**Published:** 2019-06-06

**Authors:** Sakonwan Kuhaudomlarp, Clare E. M. Stevenson, David M. Lawson, Robert A. Field

**Affiliations:** ^1^ Department of Biological Chemistry John Innes Centre, Norwich Research Park Norwich UK

**Keywords:** carbohydrate‐active enzyme, glycoside phosphorylase, oligosaccharide, surface binding site

## Abstract

Glycoside phosphorylases (GPs) with specificity for β‐(1 → 3)‐*gluco*‐oligosaccharides are potential candidate biocatalysts for oligosaccharide synthesis. GPs with this linkage specificity are found in two families thus far—glycoside hydrolase family 94 (GH94) and the recently discovered glycoside hydrolase family 149 (GH149). Previously, we reported a crystallographic study of a GH94 laminaribiose phosphorylase with specificity for disaccharides, providing insight into the enzyme's ability to recognize its' sugar substrate/product. In contrast to GH94, characterized GH149 enzymes were shown to have more flexible chain length specificity, with preference for substrate/product with higher degree of polymerization. In order to advance understanding of the specificity of GH149 enzymes, we herein solved X‐ray crystallographic structures of GH149 enzyme Pro_7066 in the absence of substrate and in complex with laminarihexaose (G6). The overall domain organization of Pro_7066 is very similar to that of GH94 family enzymes. However, two additional domains flanking its catalytic domain were found only in the GH149 enzyme. Unexpectedly, the G6 complex structure revealed an oligosaccharide surface binding site remote from the catalytic site, which, we suggest, may be associated with substrate targeting. As such, this study reports the first structure of a GH149 phosphorylase enzyme acting on β‐(1 → 3)‐*gluco*‐oligosaccharides and identifies structural elements that may be involved in defining the specificity of the GH149 enzymes.

## 1 INTRODUCTION

Research into the generation of β‐(1 → 3)‐d‐glucan polymers is of interest due to their wide range of applications such as a gelling agent in food or its use for drug encapsulation to sustain bioavailability.^1^ Enzymatic synthesis is an attractive approach to such materials due to its regiospecificity and stereospecificity. Emulating what has been achieved with α‐1,4‐glucans^2^, ^3^ and β‐1,4‐glucans,^4^, ^5^ glycoside phosphorylases (GPs)^6^, ^7^ with specificity for β‐(1 → 3)‐glycosidic bonds are potential candidate biocatalysts for such syntheses. GPs acting on β‐d‐glucopyranosyl‐(1 → 3)‐d‐glucopyranose (laminaribiose) have previously been described in bacteria *Paenibacillus* sp. YM‐1 (PsLBP)^8^ and *Acholeplasma laidlawii* PG‐8A,^9^ both of which may be found in glycoside hydrolase family 94 (GH94). We have recently investigated the activity of PsLBP, which revealed somewhat relaxed donor specificity toward a noncognate donor, α‐d‐mannose 1‐phosphate (Man1P). Our structural studies of PsLBP in complex with α‐d‐glucose 1‐phosphate (Glc1P) and Man1P revealed the architecture of the enzyme active site and the donor substrate‐1 subsite, which explain its ability to recognize the Man1P.^10^ Comparison between PsLBP and other GH94 enzyme structures revealed an additional β‐hairpin “gate” near the active site of PsLBP, which likely defines the specificity of PsLBP for disaccharide substrates/product, rather than longer oligosaccharides.

**Figure 1:**
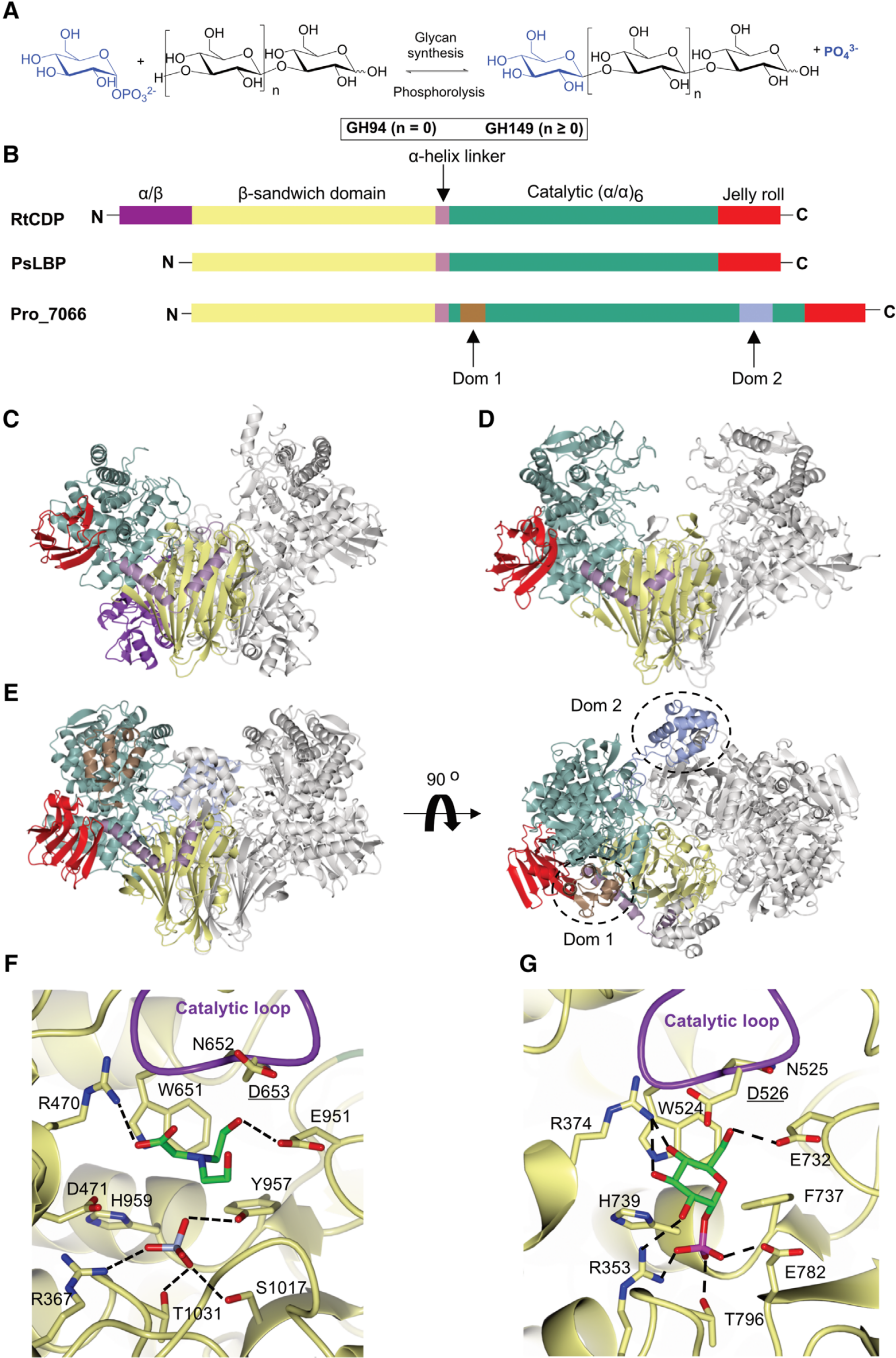
Structural comparison between RtCDP, PsLBP, and Pro_7066. A, Schematic representation of the enzymatic reaction carried out by either GH94 or GH149. B, Schematic representation of the primary sequences of RtCDP, PsLBP, and Pro_7066 colored according to the domains in the respective structures. C, Overall structures of RtCDP (PDB code; 5NZ8). D, PsLBP (PDB code; 6GH2). E, Pro_7066 structure. Additional domains in Pro_7066 (Dom 1 and Dom 2) are colored in brown and ice blue, respectively, and indicated by dotted circles. The subunit on the left is colored by domains and the right subunit in gray. F, The active site of substrate‐free Pro_7066. Omit *mFobs‐dFcalc* difference electron density maps of BCN and sulfate can be found in Figure S2. G, The active site of PsLBP in complex with Glc1P

Recently, we discovered a new glycoside hydrolase family 149 (GH149),^11^ which contains sequences from microalgae in class Euglenophyceae and notably from gram‐negative bacteria. We have characterized two enzymes from this family, one from the microalga Euglena gracilis, and the other a bacterial enzyme from a metagenomic library, Pro_7066. Both enzymes were shown to have substrate specificity for β‐(1 → 3)‐*gluco*‐oligosaccharides. Despite no significant overall sequence homology to the closely related GH94 family, multiple amino acid sequence alignments of GH149 and GH94 sequences (total of 348 sequences) revealed conservation of key amino acids that were previously identified as GH94 active site residues, suggesting a conserved catalytic mechanism between the two families of enzymes. However, the difference in chain length specificity between GH94 (acting on disaccharide substrate) and GH149 (acting on oligosaccharides) has not been investigated further (Figure 1A).

To investigate potential mechanisms underlying substrate recognition and chain length specificity in GH149 and GH94 enzymes, we solved the structure of the GH149 enzyme Pro_7066 in the absence of substrate and in complex with laminarihexaose (G6). The overall domain organization of Pro_7066 is very similar to that of GH94 family enzymes, validating the placement of both GH94 and GH149 in the glycosidehydrolase clan (GH‐Q clan). However, the Pro_7066 enzyme contains two additional unique domains flanking its catalytic domain. Comparison between the active sites of Pro_7066 and GH94 PsLBP showed a conservation of the amino acids located in the sugar phosphate donor substrate subsite, consistent with predictions from multiple sequence alignments. Unexpectedly, the G6 complex structure did not reveal occupancy of the acceptor substrate site, but instead an additional oligosaccharide surface binding site (SBS) on the catalytic domain of the enzyme, which we speculate may be associated with substrate targeting. This study reports the first structure of a GH149 phosphorylase enzyme acting on β‐(1 → 3)‐*gluco*‐oligosaccharides and provides insight into substrate binding.

## 2 MATERIALS AND METHODS

### 2.1 Production of Pro_7066 recombinant protein

The nucleotide sequence of Pro_7066 (Prozomix Limited) was cloned into the pET28a vector and transformed into chemically competent *Escherichia* coli BL21 (DE3) cells. Transformants were selected on LB agar containing 50 μg/mL kanamycin and grown at 22°C overnight in 1 L terrific broth containing the same antibiotic. Expression was induced with the addition of Isopropyl β‐D‐1‐thiogalactopyranoside (IPTG) to the final concentration of 0.2 mM and the culture was further incubated overnight at 18°C. Cells were harvested by centrifugation (7000*g*, 10 minutes) and lysed by sonication in buffer A (10 mM 4‐(2‐hydroxyethyl)‐1‐piperazineethanesulfonic acid [HEPES] pH 7.5 and 250 mM NaCl) supplemented with 1 mg/mL DNase (Sigma) and one tablet of a complete protease inhibitor cocktail (Roche). Supernatant containing the recombinant proteins was separated from cell debris by centrifugation (33 000*g*, 30 minutes). Proteins were purified with an ÄKTA pure fast protein liquid chromatography (FPLC) system (GE Healthcare) at 4°C. The supernatant containing His_6_‐tagged recombinant protein was loaded to a 1‐mL HisTrap HP column (GE Healthcare) preequilibrated with buffer A. The column was washed with buffer A and bound proteins were eluted in one step with buffer B (10 mM HEPES pH 7.5, 250 mM NaCl, and 500 mM imidazole). The proteins were further purified by gel filtration using a Superdex S200 16/600 column (GE Healthcare), eluted with 20 mM HEPES pH 7.5, 150 mM NaCl, at 1 mL/min. Fractions containing protein were pooled and concentrated to 10 mg/mL using an Amicon Ultra‐15 30 kDa molecular weight cut off concentrator. The purified Pro_7066 protein was subsequently stored in 30 μL aliquots at −80°C until required.

### 2.2 Crystal structure determination

Crystallization trials were set up for purified Pro_7066 (∼15 mg/mL in 20 mM HEPES pH 7.0 and 150 mM NaCl) using the Basic Chemical Space (BCS) HT‐96 screen (Molecular Dimensions) in Medical Research Council (MRC) 2‐drop 96‐well sitting‐drop vapor diffusion crystallization plates (Swissi) with a mixture of 0.3 μL well solution and 0.3 μL protein solution in drop 1 and the same conditions was applied in drop 2 with an addition of 8 mM laminarihexaose (G6) (Megazyme) using an OryxNano robot (Douglas Instruments). The best crystallization hit was detected in the well containing 0.2 M ammonium sulfate, 0.05 M magnesium sulfate heptahydrate, 0.1 M bicine (BCN) pH 9.0, and 20% (v/v) polyethylene glycol (PEG) Smear Medium (Molecular Dimensions). These conditions were then optimized (19.5‐20.5% PEG Smear Medium [Molecular Dimensions], 0.15‐0.25 M ammonium sulfate, 0.045‐0.055 magnesium sulfate heptahydrate, and 0.1 M BCN, pH 9.0) to obtain better quality crystals. The best crystals obtained from the optimization were cryoprotected with well solution containing an additional 20% (v/v) ethylene glycol and flash‐cooled in liquid nitrogen. For phasing, crystals were soaked for 30 minutes in a saturated solution of mercury (II) chloride made up in the cryoprotectant solution.

The precooled crystals were transferred robotically to the goniostat on either beamline I03 or I04 at Diamond Light Source (Oxfordshire, UK) and maintained at −173°C with a Cryojet cryocooler (Oxford Instruments). X‐ray diffraction data were recorded using a Pilatus 6 M hybrid photon counting detector (Dectris), then integrated, and scaled using X‐ray Detector Software (XDS)^12^ via the XIA2 expert system^13^ and merged using AIMLESS^14^ All crystals belonged to space group P2_1_2_1_2_1_ with approximate cell parameters of *a* = 100.2, *b* = 159.0, and *c* = 181.6 Å.

Analysis of the likely composition of the asymmetric unit (ASU) suggested that it would contain two copies of the 130‐kDa protein chain, giving an estimated solvent content of 55%. The structure was solved at 2.6 Å resolution by single‐wavelength anomalous dispersion (SAD) phasing using the CRANK2 pipeline^15^ with data collected from a mercury‐soaked crystal at the L_III_ X‐ray absorption edge of mercury (wavelength = 1.0052 Å). SHELXD^16^ located 17 sites in the ASU with occupancies >0.25 and BUCCANEER^17^ went on to build a model in which 97% of the sequence was fitted with *R*_work_ and *R*_free_ values of 0.288 and 0.340, respectively. This was then edited in COOT^18^ before refining in REFMAC5^19^ against native data processed to 2.05 Å resolution. The model was finalized by further iterations of manual rebuilding in COOT and restrained refinement in REFMAC5 using isotropic thermal parameters and Translation‐libration‐screw‐rotation (TLS) group definitions obtained from the TLSMD server (http://skuld.bmsc.washington.edu/∼tlsmd/).^20^ In each of the expected active sites, residual density consistent with sulfate and BCN derived from the precipitant solution was present. This structure is referred to as the substrate‐free complex and was used as the starting point for the G6 which was refined using a similar protocol. The geometries of the final models were validated with MOLPROBITY^21^ and the Worldwide Protein Data Bank (wwPDB) validation service (https://validate-rcsb-1.wwpdb.org/) before submission to the PDB. Omit *mFobs‐dFcalc* difference electron density maps were generated for the selected ligands using phases from the final model without the ligands after the application of small random shifts to the atomic coordinates, resetting temperature factors, and rerefining to convergence. All structural figures were prepared using CCP4MG.^22^ Data collection and processing statistics for both the Pro_7066 structures are summarized in Table 1.

**Table 1:** X‐ray data collection and refinement of Pro_7066 structures

Data set	Mercury derivative	Substrate‐free complex	G6 complex
Data collection			
Beamline	I04	I04	I04
Wavelength (Å)	1.0052	0.9795	0.9795
Detector	Pilatus 6M	Pilatus 6M	Pilatus 6M
Resolution range (Å)^a^	62.26‐2.55 (2.59‐2.55)	79.49‐2.05 (2.09‐2.05)	66.41‐2.25 (2.29‐2.25)
Space group	P2_1_2_1_2_1_	P2_1_2_1_2_1_	P2_1_2_1_2_1_
*a*, *b*, and *c* (Å)	99.8, 159.3, and 180.9	100.2, 159.0, and 181.6	99.1, 158.8, and 178.9
*α*, *β*, and *γ* (°)	90.0, 90.0, and 90.0	90.0, 90.0, and 90.0	90.0, 90.0, and 90.0
Total observations^a^	2 559 459 (127 918)	2 447 545 (123 380)	1 808 389 (88 317)
Unique reflections^a^	94 654 (4584)	181 781 (8926)	134 201 (6512)
Multiplicity^a^	27.0 (27.9)	13.5 (13.8)	13.5 (13.6)
Mean *I*/σ (*I*)^a^	11.3 (1.4)	14.7 (1.1)	13.5 (1.4)
Completeness (%)^a^	100.0 (100.0)	100.0 (100.0)	100.0 (100.0)
*R*_merge_ a ^,^ b	0.240 (2.758)	0.107 (2.387)	0.132 (2.024)
*R*_meas_ a ^,^ c	0.244 (2.808)	0.111 (2.478)	0.138 (2.103)
CC_½_ a ^,^ d	.998 (.741)	.999 (.549)	.999 (.637)
Wilson *B* value (Å^2^)	44.2	41.4	45.0
Refinement			
Reflections: working/free^e^	‐	172 561/9118	127 355/6752
*R*_work_/*R*_free_ f	‐	0.182 (0.292)/0.210 (0.322)	0.180 (0.278)/0.217 (0.296)
Ramachandran plot: favored/allowed/disallowed (%)^g^	‐	97/3/0	96/4/0
RMSZ bond^g^	‐	0.53	0.50
RMSZ angle^g^	‐	0.64	0.64
No. of protein residues: chain A/chain B	‐	1135/1133	1138/1138
No. of ligands^h^/water molecules	‐	27/865	21/699
Mean *B* factors: protein/ligands^h^//water/overall (Å^2^)	‐	64/61/48/63	60/63/47/60
PDB accession code	‐	6HQ6	6HQ8

Abbreviation: PDB, Protein Data Bank; RMSZ, The root‐mean‐square value of the Z‐scores of bond lengths (or angles).

a Values for the outer resolution shell are given in parentheses.

b *R*_merge_ = ∑_*hkl*_∑_*i*_ ∣ *I*_*i*_(*hkl*) – 〈*I*(*hkl*)〉 ∣ /∑_*hkl*_∑_*i*_*I*_*i*_(*hkl*).

c *R*_meas_ = ∑_*hkl*_[*N*/(*N* − 1)]^1/2^ × ∑_*i*_ ∣ *I*_*i*_(*hkl*) − 〈*I*(*hkl*)〉 ∣ /∑_*hkl*_∑_*i*_*I*_*i*_(*hkl*), where *I*_*i*_(*hkl*) is the *i*th observation of reflection *hkl*, 〈*I*(*hkl*)〉 is the weighted average intensity for all observations *i* of reflection *hkl* and *N* is the number of observations of reflection *hkl*.

d CC_½_ is the correlation coefficient between symmetry‐related intensities taken from random halves of the dataset.

e The data set was split into “working” and “free” sets consisting of 95% and 5% of the data, respectively. The free set was not used for refinement.

f The *R* factors *R*_work_ and *R*_free_ are calculated as follows: *R* =  ∑ (| *F*_obs_ − *F*_calc_| )/ ∑ |*F*_obs_|, where *F*_obs_ and *F*_calc_ are the observed and calculated structure factor amplitudes, respectively.

g PDB validation reports.

h This includes sulfate, bicine, G6, chloride ions, and ethylene glycol molecules.

## 3 RESULTS AND DISCUSSION

### 3.1 Overall structure of Pro_7066

X‐ray crystallography was used to determine the structure of Pro_7066: the substrate‐free structure was solved to 2.05 Å resolution. Structures with bound oligosaccharide ligands were then sought through co‐crystallization with laminaribiose (G2), laminaritriose (G3), laminaritriosetetraose (G4), laminaritriosepentaose (G5) or laminaritriosehexaose (G6) (at 8 mM). However, useful data could only be obtained from the G6 co‐crystallization, the structure of which was solved to 2.25 Å resolution. Both structures belong to space group P2_1_2_1_2_1_ and contain two subunits per ASU. The two subunits superposed with an root‐mean‐square deviation of atomic positions (RMSD) of 0.23 Å. The two copies of the molecule in the ASU form a biologically relevant homodimer with an interfacial area of ∼4230 Å^2^ were calculated by jsPISA.^23^ The formation of a homodimer in the crystal structure is consistent with the gel filtration analysis, where Pro_7066 was eluted as a dimer.^11^ The domains present within each subunit can be defined as follows: an N‐terminal β‐sandwich (residues 1‐303, yellow), a helical linker region (residues 304‐341, lilac), an (α/α)_6_ catalytic domain (residues 342‐1045, green) and a C‐terminal jelly roll domain (residues 1046‐1156, red) (Figure 1B). These domains are similar to those observed in PsLBP^10^ and other GH94 enzymes (Figure 1C,D).^5^, ^24^, ^25^, ^26^, ^27^, ^28^ Cellodextrin phosphorylase from *Ruminiclostridium thermocellum* (RtCDP)^5^ and β‐(1 → 2)‐oligoglucan phosphorylase from *Lachnoclostridium phytofermentans* (LpSOGP),^27^ both belonging to GH94 family, have additional, but dissimilar, N‐terminal domains (Figure 1C, purple in RtCDP), but neither is present in Pro_7066.

Interestingly, there are two extra domains inserted within the catalytic domain of Pro_7066, designated Dom 1 (residues 370‐428, brown) and Dom 2 (residues 852‐938, ice blue) (Figure 1E). The structures of Dom 1 and Dom 2 were investigated further by Distance‐matrix Alignment (DALI) analysis^29^ (http://ekhidna2.biocenter.helsinki.fi/dali/), which showed that Dom 1 and Dom 2 are not significantly similar to domains in any known protein structures according to the DALI analysis (*Z* scores < 5). Analysis of Dom 1 and Dom 2 sequences by Pfam^30^ and an NCBI‐conserved domain search^31^ did not reveal any significant hits.

### 3.2 Pro_7066 active site

The architecture of the Pro_7066 active site is highly similar to that observed for GH94 enzymes (Figure 1F,G). The conserved structural characteristics include (a) the presence of a tryptophan ‐ asparagine ‐ aspartate (WND) motif (W651, N652, and D653) in the catalytic loop, with D653 as the predicted catalytic residue, although the D653 side chain is rotated 100° around Cα—Cβ bond in comparison to that in PsLBP structure; (b) a conserved arginine ‐ aspartate (RD) motif (R470 and D471), which is involved in the recognition of sugar 1‐phosphate, although in the PsLBP structure, the distance between the aspartate residue (D375) and Glc1P is greater than the hydrogen bonding distance; (c) the conserved histidine (H959), which was predicted to be involved in phosphate recognition,^25^ although this residue does not form hydrogen bonds with the sulfate molecule in Pro_7066 or PsLBP structures. These structurally conserved amino acids are in agreement with the sequence alignment which predicted the presence of these residues in the enzyme active site.^11^ Bicine (BCN) derived from the crystallization precipitant was bound via hydrogen bonding contacts with R470 and E951, possibly mimicking the interaction of the enzyme active site with the hydroxy groups on C3 and C6 of Glc1P donor (Figure 1F). A sulfate molecule was observed (ice blue, Figure 1F) occupying a similar position to the phosphate moiety of Glc1P in the PsLBP structure (magenta, Figure 1G).

In order to rationalize the difference in substrate preferences between PsLBP and Pro_7066, the structures of PsLBP (PDB code 6GH2) and substrate‐free Pro_7066 (PDB code 6HQ6) were superimposed. Although the RMSD values suggest that these structures are not highly similar (RMSD > 1 Å), the structural elements neighboring the active site are relatively well conserved (Figure S1): (a) the gate (blue) described in PsLBP can be identified in Pro_7066, although in the latter it is part of a disordered loop; (b) the opposing (red) and the adjacent loops (brown), which are present in all of the three structures. Despite the structural conservation, there is no conservation in the amino acid compositions of these loops. In the Pro_7066 structure, Dom 2 (Figure S1, green) is found in proximity to the gate. The average B‐factor of Dom 2 (∼70 Å^2^) is higher than the average B‐factor of the protein subunit (48 Å^2^), suggesting Dom 2 is more dynamic compared to the rest of the subunit.

As well as the conserved structural elements, there is an additional feature, designated as an “upper” strand (Figure S1, pink). This feature is located above the adjacent loop in PsLBP (residue 465‐480) and Pro_7066 (residue 568‐603). In Pro_7066, the upper strand is 20‐residue longer than that in PsLBP and contains a helical structure connected to a small β‐hairpin, whereas the portion connected to the hairpin is disordered in PsLBP.

### 3.3 Structure of Pro_7066 in complex with G6 revealed an oligosaccharide SBS on the catalytic domain

**Figure 2:**
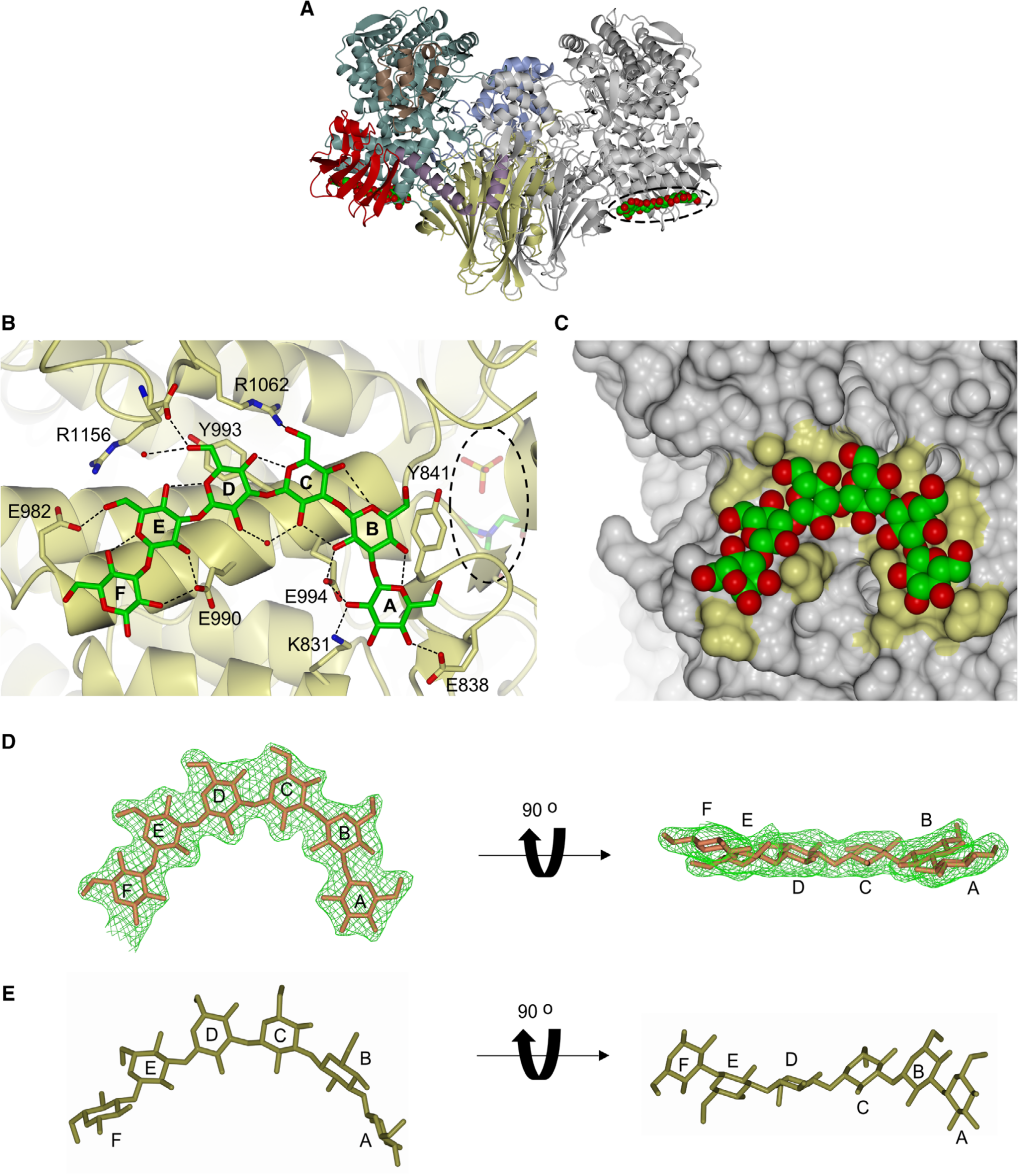
The SBS site on the surface of Pro_7066 catalytic domain. A, Pro_7066 structure in complex with G6. The location of G6 is indicated by dotted oval. B, SBS site with G6 bound. The enzyme backbone is presented as ribbon (yellow). The active site with BCN and ${\mathrm{SO}}_{4}^{2-}$ bound is buried within the catalytic domain and is on the opposite site of the SBS (dotted oval). Carbon atoms of G6 are presented in green and oxygen in red. The Glc residues in G6 are designated as *A*‐*F* from the nonreducing to the reducing end. C, Molecular surface representation of the SBS, with yellow patches representing the location of side chains that are involved in the interaction with G6. G6 is represented in space‐filled model. D, G6 ligand at the SBS of Pro_7066 with the omit *mFobs‐dFcalc* difference electron density (green mesh). E, Free G6 structure built by Polys Glycan Builder (http://glycan‐builder.cermav.cnrs.fr) with *ϕ*/*ψ* torsion angles = −72°/108°. SBS, surface binding site

The overall structure of the G6 complex is similar to the substrate‐free complex (RMSD = 0.35 Å for a dimer on dimer superposition). The active site of the G6 complex is occupied by BCN and ${\mathrm{SO}}_{4}^{2-}$, identical to that in the substrate‐free complex. There was no detectable binding of G6 within the active site. However, G6 bound to a SBS located on the solvent‐exposed surface of the catalytic domain and is ∼30 Å away from the active site (Figure 2A,B). Although structures with G2‐G5 bound could not be satisfactorily refined due to the poor quality of the diffraction data, the autoprocessed data from the G2‐G5 co‐crystallizations were analyzed by Dimple,^32^ which showed weak residual electron density consistent with sugar pyranose rings in the same SBS site.

The SBS consists of two aromatic residues (Y841 and Y993) that form stacking interactions with sugar residues B and D of G6, respectively. Several hydrogen bonds are formed between the hydroxy groups (mainly C2—OH) of G6 and the SBS amino acid side chains (K831, E838, E990, E994 E982, and R1062) (Figures 2B and S3B). Comparison between the G6 and substrate‐free complexes revealed dynamic conformations of the side chains that make hydrogen bonding contacts with G6 (E838, E982, E990, Y993, and E994) (Figure S3A). Molecular surface representation of the SBS (Figure 2C) revealed a shallow cleft with a U‐shaped topology, which complements the curvature of G6, the conformation commonly adopted by β‐(1 → 3)‐*gluco*‐oligosaccharides.^33^, ^34^

Molecular dynamics (MD) simulations of β‐(1 → 3)‐*gluco*‐oligosaccharides performed by Frecer et al^33^ revealed the global energy minima of the oligosaccharide is at *φ*/*ψ* torsion angles −72°/108°, and a triple‐helical conformation is the most favorable with pitch 22 Å and six Glc residues per turn, suggesting that free G6 is capable of adopting a conformation similar to that found in the β‐(1 → 3)‐glucan triple helical structure in solution.^35^ The structure of free G6 based on the calculated MD torsion angles was built in Polys Glycan Builder (http://glycan-builder.cermav.cnrs.fr/) and compared with the conformation of the G6 ligand in complex with Pro_7066, which showed that the G6 ligand is relatively flat compared to the free G6 structure that is more twisted at residues A, B, E, and F (Figure 2D,E). The triple helical structure of β‐(1 → 3)‐glucan is stabilized by interstrand and intrastrand hydrogen bonds. The interstrand hydrogen bonds are formed between OH groups on C2 (C2—OH),^36^ while intrastrand hydrogen bonds are formed between O4 and O5 of adjacent Glc subunits.^36^, ^37^, ^38^ In the Pro_7066 G6 complex, the C2—OH groups are predominantly involved in the interaction with the SBS.

To determine whether the SBS observed in Pro_7066 is present in GH94 enzymes or in other GH149 sequences, multiple alignment of amino acid sequences from GH94 and GH149 families was performed, with the focus on the conservation of E990, Y993, and E994, which form intimate contacts with G6 in Pro_7066. The alignment showed that the amino acids found in Pro_7066 SBS are not 100% conserved in GH94 enzymes (Figure S4). Among GH149 sequences alone, the sequence alignment revealed that Y993 and E994 (tyrosine ‐ glutamate [YE] motif) are relatively conserved in Euglenophyceae sequences, with two of the *Eutreptiella* sequences (200418968 and 200416338) having the glutamate substituted by aspartate, while E990 is less conserved (Figure S4). In contrast, the YE motif and E990 are relatively conserved in the bacterial GH149 sequences that shared >60% overall sequence identity to Pro_7066, with an exception to WP_035128081.1 from Flavobacterium aquatile in which YE motif and E990 are not conserved. The YE motif and E990 are less conserved in the other bacterial GH149 sequences that shared lower sequence identity to Pro_7066.

In summary, despite their relatively low amino acid sequence similarities, we provide the first structural evidence for conserved catalytic folds among enzymes from the GH94 and GH149 families, which validate their placement in the same GH‐Q clan. We have also uncovered additional structural elements in the GH149 enzyme, in particular the unique SBS which may contribute to the ability of the enzyme to operate on longer oligosaccharide substrates in comparison to GH94 PsLBP.

## CONFLICT OF INTERESTS

The authors declare no potential conflict of interest.

## Supporting information


**Data S1** Supporting information

**Data S2** Supporting information

**Data S3** Supporting information

## References

[1] Laroche C , Michaud P . New developments and prospective applications for β(1,3) glucans. Recent Pat Biotechnol. 2007;1:59‐73. 19075833 10.2174/187220807779813938

[2] O'Neill EC , Rashid AM , Stevenson CEM , et al. Sugar‐coated sensor chip and nanoparticle surfaces for the *in vitro* enzymatic synthesis of starch‐like materials. Chem Sci. 2014;5:341‐350.

[3] O'Neill EC , Field RA . Underpinning starch biology with in vitro studies on carbohydrate‐active enzymes and biosynthetic glycomaterials. Front Bioeng Biotechnol. 2015;3:1‐6. 26442250 10.3389/fbioe.2015.00136 PMC4561517

[4] Petrović DM , Kok I , Woortman AJJ , Ćirić J , Loos K . Characterization of oligocellulose synthesized by reverse phosphorolysis using different cellodextrin phosphorylases. Anal Chem. 2015;87:9639‐9646. 26291473 10.1021/acs.analchem.5b01098

[5] O'Neill EC , Pergolizzi G , Stevenson CEM , Lawson DM , Nepogodiev SA , Field RA . Cellodextrin phosphorylase from *Ruminiclostridium thermocellum*: X‐ray crystal structure and substrate specificity analysis. Carbohydr Res. 2017;451:118‐132. 28760417 10.1016/j.carres.2017.07.005 PMC5667895

[6] O'Neill EC , Field RA . Enzymatic synthesis using glycoside phosphorylases. Carbohydr Res. 2015;403:23‐37. 25060838 10.1016/j.carres.2014.06.010 PMC4336185

[7] Pergolizzi G , Kuhaudomlarp S , Kalita E , Field RA . Glycan phosphorylases in multi‐enzyme synthetic processes. Protein Pept Lett. 2017;24:696‐709. 28799504 10.2174/0929866524666170811125109 PMC5688430

[8] Kitaoka M , Matsuoka Y , Mori K , Nishimoto M , Hayashi K . Characterization of a bacterial laminaribiose phosphorylase. Biosci Biotechnol Biochem. 2012;76:343‐348. 22313784 10.1271/bbb.110772

[9] Nihira T , Saito Y , Kitaoka M , Nishimoto M , Otsubo K , Nakai H . Characterization of a laminaribiose phosphorylase from *Acholeplasma laidlawii* PG‐8A and production of 1,3‐β‐d‐glucosyl disaccharides. Carbohydr Res. 2012;361:49‐54. 22982171 10.1016/j.carres.2012.08.006

[10] Kuhaudomlarp S , Walpole S , Stevenson CEM , et al. Unravelling the specificity of laminaribiose phosphorylase from *Paenibacillus* sp. YM‐1 towards donor substrates glucose/mannose 1‐phosphate by using X‐ray crystallography and saturation transfer difference NMR spectroscopy. Chembiochem. 2018;20:181‐192. 29856496 10.1002/cbic.201800260

[11] Kuhaudomlarp S , Patron NJ , Henrissat B , Rejzek M , Saalbach G , Field RA . Identification of Euglena gracilis β‐1,3‐glucan phosphorylase and establishment of a new glycoside hydrolase (GH) family GH149. J Biol Chem. 2018;293:2865‐2876. 29317507 10.1074/jbc.RA117.000936 PMC5827456

[12] Kabsch W . XDS. Acta Crystallogr Sect D. 2010;66:125‐132. 20124692 10.1107/S0907444909047337 PMC2815665

[13] Winter G . Xia2: an expert system for macromolecular crystallography data reduction. J Appl Crystallogr. 2010;43:186‐190.

[14] Evans PR , Murshudov GN . How good are my data and what is the resolution? Acta Crystallogr Sect D Biol Crystallogr. 2013;69:1204‐1214. 23793146 10.1107/S0907444913000061 PMC3689523

[15] Skubák P , Pannu NS . Automatic protein structure solution from weak X‐ray data. Nat Commun. 2013;4:2777. 24231803 10.1038/ncomms3777 PMC3868232

[16] Sheldrick GM . A short history of SHELX. Acta Crystallogr Sect A Found Crystallogr. 2007;64:112‐122. 10.1107/S0108767307043930 18156677

[17] Cowtan K . The *Buccaneer* software for automated model building. 1. Tracing protein chains. Acta Crystallogr Sect D. 2006;62:1002‐1011. 16929101 10.1107/S0907444906022116

[18] Emsley P , Cowtan K . *Coot*: model‐building tools for molecular graphics. Acta Crystallogr Sect D. 2004;60:2126‐2132. 15572765 10.1107/S0907444904019158

[19] Murshudov GN , Vagin AA , Dodson EJ . Refinement of macromolecular structures by the maximum‐likelihood method. Acta Crystallogr Sect D. 1997;53:240‐255. 15299926 10.1107/S0907444996012255

[20] Painter J , Merritt EA . *TLSMD* web server for the generation of multi‐group TLS models. J Appl Crystallogr. 2006;39:109‐111.

[21] Davis IW , Leaver‐Fay A , Chen VB , et al. MolProbity: all‐atom contacts and structure validation for proteins and nucleic acids. Nucleic Acids Res. 2007;35:W375‐W383. 17452350 10.1093/nar/gkm216 PMC1933162

[22] McNicholas S , Potterton E , Wilson KS , Noble MEM . Presenting your structures: the CCP4mg molecular‐graphics software. Acta Crystallogr Sect D Biol Crystallogr. 2011;67:386‐394. 21460457 10.1107/S0907444911007281 PMC3069754

[23] Krissinel E . Stock‐based detection of protein oligomeric states in jsPISA. Nucleic Acids Res. 2015;43:W314‐W319. 25908787 10.1093/nar/gkv314 PMC4489313

[24] Van Hoorebeke A , Stout J , Kyndt J , et al. Crystallization and X‐ray diffraction studies of cellobiose phosphorylase from *Cellulomonas uda* . Acta Crystallogr Sect F Struct Biol Cryst Commun. 2010;66:346‐351. 10.1107/S1744309110002642 PMC2833054 20208178

[25] Hidaka M , Kitaoka M , Hayashi K , Wakagi T , Shoun H , Fushinobu S . Structural dissection of the reaction mechanism of cellobiose phosphorylase. Biochem J. 2006;398:37‐43. 16646954 10.1042/BJ20060274 PMC1525018

[26] Nam Y‐W , Nihira T , Arakawa T , et al. Crystal structure and substrate recognition of cellobionic acid phosphorylase, which plays a key role in oxidative cellulose degradation by microbes. J Biol Chem. 2015;290:18281‐18292. 26041776 10.1074/jbc.M115.664664 PMC4513089

[27] Nakajima M , Tanaka N , Furukawa N , et al. Mechanistic insight into the substrate specificity of 1,2‐β‐oligoglucan phosphorylase from *Lachnoclostridium phytofermentans* . Sci Rep. 2017;7:42671. 28198470 10.1038/srep42671 PMC5309861

[28] Hidaka M , Honda Y , Kitaoka M , et al. Chitobiose phosphorylase from *Vibrio proteolyticus*, a member of glycosyl transferase family 36, has a clan GH‐l‐like (α/α)(6) barrel fold. Structure. 2004;12:937‐947. 15274915 10.1016/j.str.2004.03.027

[29] Holm L , Laakso LM . Dali server update. Nucleic Acids Res. 2016;44:W351‐W355. 27131377 10.1093/nar/gkw357 PMC4987910

[30] Finn RD , Coggill P , Eberhardt RY , et al. The Pfam protein families database: towards a more sustainable future. Nucleic Acids Res. 2016;44:D279‐D285. 26673716 10.1093/nar/gkv1344 PMC4702930

[31] Marchler‐Bauer A , Bo Y , Han L , et al. CDD/SPARCLE: functional classification of proteins via subfamily domain architectures. Nucleic Acids Res. 2017;45:D200‐D203. 27899674 10.1093/nar/gkw1129 PMC5210587

[32] Keegan R , Wojdyr M , Winter G , Ashton A . DIMPLE: a difference map pipeline for the rapid screening of crystals on the beamline. Acta Crystallogr Sect A Found Adv. 2015;71:s18.

[33] Frecer V , Rizzo R , Miertus S . Molecular dynamics study on the conformational stability of laminaran oligomers in various solvents. Biomacromolecules. 2000;1:91‐99. 11709848 10.1021/bm990002f

[34] Boraston AB , Nurizzo D , Notenboom V , et al. Differential oligosaccharide recognition by evolutionarily‐related β‐1,4 and β‐1,3 glucan‐binding modules. J Mol Biol. 2002;319:1143‐1156. 12079353 10.1016/S0022-2836(02)00374-1

[35] Vasur J , Kawai R , Jonsson KHM , et al. Synthesis of cyclic β‐glucan using laminarinase 16a glycosynthase mutant from the Basidiomycete *Phanerochaete chrysosporium* . J am Chem Soc. 2010;132:1724‐1730. 20078120 10.1021/ja909129b

[36] Miyoshi K , Uezu K , Sakurai K , Shinkai S . Proposal of a new hydrogen‐bonding form to maintain curdlan triple helix. Chem Biodivers. 2004;1:916‐924. 17191891 10.1002/cbdv.200490073

[37] Takahasi K , Ochiai M , Horiuchi M , et al. Solution structure of the silkworm betaGRP/GNBP3 N‐terminal domain reveals the mechanism for beta‐1,3‐glucan‐specific recognition. Proc Natl Acad Sci. 2009;106:11679‐11684. 19561300 10.1073/pnas.0901671106 PMC2710696

[38] Qin Z , Yang D , You X , et al. The recognition mechanism of triple‐helical β‐1,3‐glucan by a β‐1,3‐glucanase. Chem Commun. 2017;53:9368‐9371. 10.1039/c7cc03330c 28787048

