# Supplementary material for: The structure of a GH149 β‐(1 → 3) glucan phosphorylase reveals a new surface oligosaccharide binding site and additional domains that are absent in the disaccharide‐specific GH94 glucose‐β‐(1 → 3)‐glucose (laminaribiose) phosphorylase
Source: Proteins. 2019 Jun 6;87(10):885–92. doi: 10.1002/prot.25745 (PMC6771811; doi:10.1002/prot.25745)
Supplement: Supplementary file 1 — Data S1 Supporting information [file PROT-87-885-s001.pdf]

Supplementary information

The structure of a GH149 β-(1→3) glucan phosphorylase reveals a new surface oligosaccharide binding site and additional domains that are absent in the disaccharide-specific GH94 glucose-β-(1→3)-glucose (laminaribiose) phosphorylase

Sakonwan Kuhaudomlarp,[a,b] Clare E. M. Stevenson,[a] David M. Lawson,[a] Robert A. Field[a,*]

[a] Department of Biological Chemistry, John Innes Centre, Norwich Research Park, Norwich, NR4 7UH, UK;
[b] Present address: Univ. Grenoble Alpes, CNRS, CERMAV, 38000 Grenoble, France.

*Correspondence to: Robert A. Field, Department of Biological Chemistry, John Innes Centre, Norwich Research Park, Norwich, NR4 7UH, UK
E-mail: rob.field@jic.ac.uk

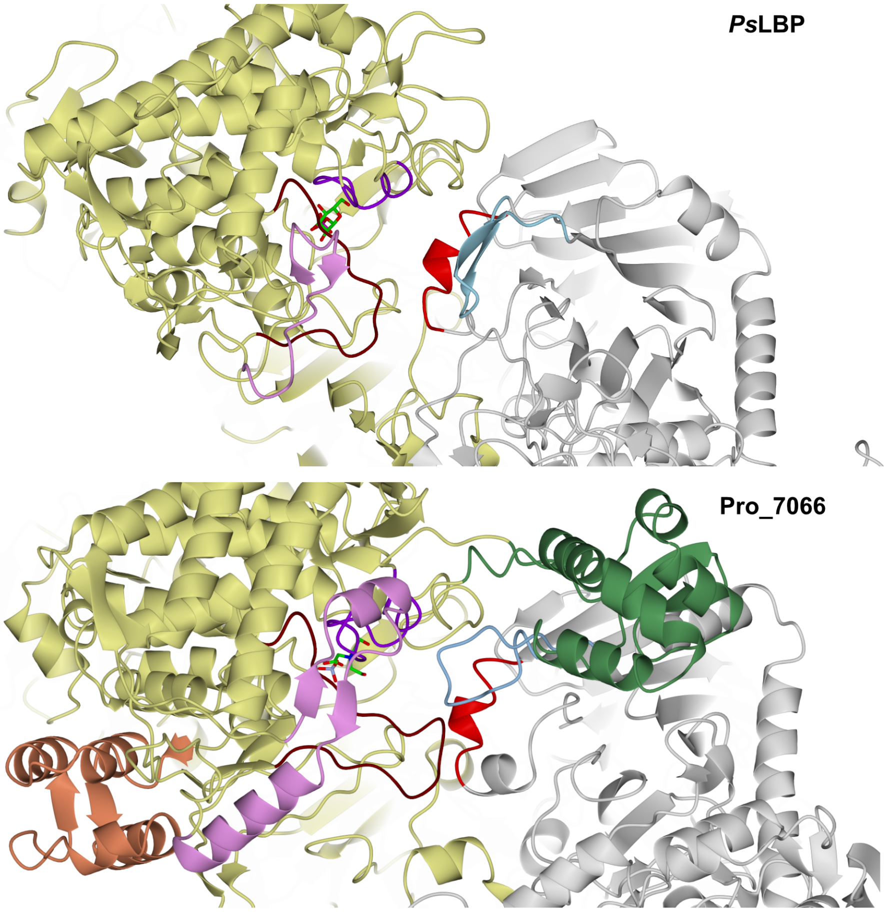

Figure S1. Structural elements surrounding the active sites of PsLBP and Pro_7066. Purple = catalytic loop, red = opposing loop, blue = gate, brown = adjacent loop, pink = upper strand, orange = Dom 1, green = Dom 2. The gate and opposing loops are located in the adjacent subunit.

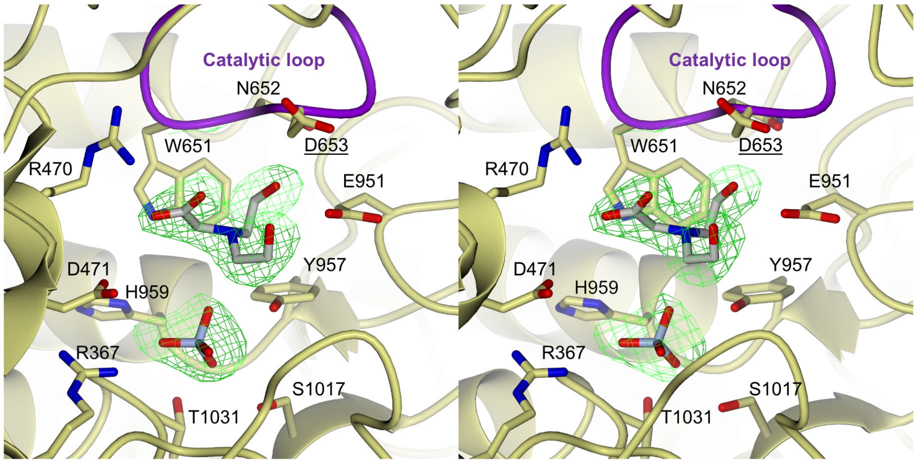

Figure S2. Stereo view of the Pro_7066 active site. Omit *mFobs-dFcalc* difference electron density maps (2.05 Å resolution; contoured at ~3σ) shown in green mesh and generated for the BCN and sulfate.

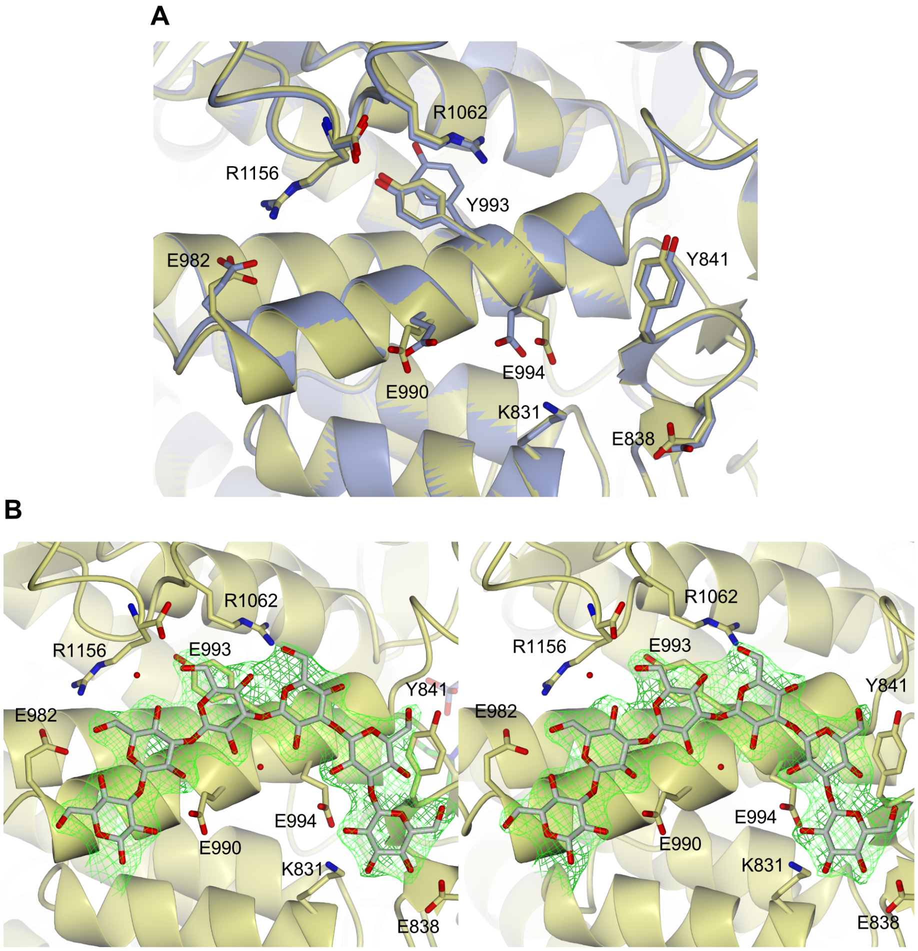

Figure S3. (**A**) Superposition of the SBS site in the G6 complex (yellow) with the substrate-free complex (blue). Electron density indicates the presence of Y993 side chain in two alternate conformations in the substrate-free complex. (**B**) A stereo image of the SBS with an omit *mFobs-dFcalc* difference electron density map (2.25 Å resolution; contoured at ~3σ) shown in green mesh, which was generated for the bound ligand using phases calculated from the final model without the ligand after the application of small random shifts to the atomic coordinates, re-setting temperature factors, and re-refining to convergence.

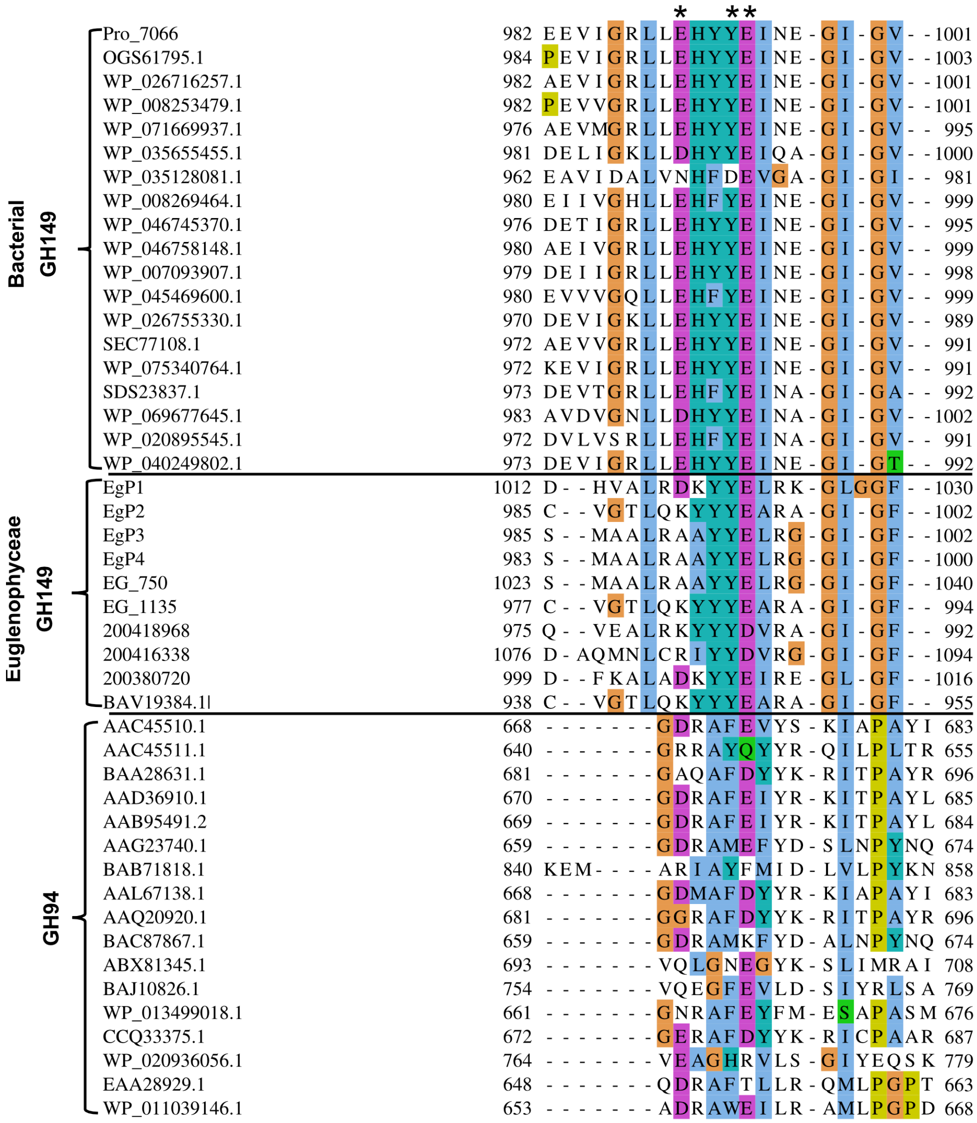

Figure S4. Multiple amino acid sequence alignment of Pro_7066 with Euglenophyceae GH149, bacterial GH149 and characterised bacterial GH94 sequences. The presented bacterial GH149 sequences shared >60% sequence identity to Pro_7066. The amino acids are coloured according to Clustal amino acid colour scheme. Asterisks indicate the amino acids that are in intimate contacts with G6 in Pro_7066. The organism sources of the sequences are as followed; OGS61795.1 [*Flavobacteria bacterium* GWF1_32_7], WP_026716257.1 [*Flavobacterium gelidilacus*], WP_008253479.1 [*Flavobacteria bacterium* BAL38], WP_071669937.1 [*Flavobacteriaceae* bacterium UJ101], WP_035655455.1 [*Flavobacterium filum*], WP_035128081.1 [*Flavobacterium aquatile*], WP_008269464.1 [*Flavobacteriales* bacterium ALC-1], WP_046745370.1 [*Kordia zhangzhouensis*], WP_046758148.1 [*Kordia jejudonensis*], WP_007093907.1 *[Kordia algicida*], WP_045469600.1 [*Winogradskyella* sp. PG-2], WP_026755330.1 [*Sediminibacter* sp. Hel_I_10], SEC77108.1 [*Tenacibaculum* sp. MAR_2009_124], WP_075340764.1 [*Tenacibaculum* sp. HZ1], SDS23837.1 [*Formosa* sp. Hel1_31_208], WP_069677645.1 [*Formosa* sp. Hel1_33_131], WP_020895545.1 [*Winogradskyella psychrotolerans*], WP_040249802.1 [*Psychroserpens mesophilus*], EgP1-4 [*Euglena gracilis*], EG_750 [*Euglena gracilis*], EG_1135 [*Euglena gracilis*], BAV19384.1 [*Euglena gracilis*], 200418968 [*Eutreptiella gymnastica*], 200416338 [*Eutreptiella gymnastica*], 200380720 [*Eutreptiella gymnastica*], AAC45510.1 [*Clostridium stercorarium*], AAC45511.1

[*Clostridium stercorarium*], BAA28631.1 [*Cellvibrio gilvus* ATCC13127], AAD36910.1 [*Thermotoga maritima*], AAB95491.2 [*Thermotoga neapolitana*], AAG23740.1 [*Vibrio furnissii*], BAB71818.1 [*Ruminiclostridium thermocellum*], AAL67138.1 [*Ruminiclostridium thermocellum*], AAQ20920.1 [*Cellulomonas uda*], BAC87867.1 [*Vibrio proteolyticus*], ABX81345.1 [*Acholeplasma laidlawii* PG-8A], BAJ10826.1 [*Paenibacillus* sp. YM1], WP_013499018.1 [*Ruminococcus albus*], CCQ33375.1 [*Halorhapdus tiamatea* SARL4B], WP_020936056.1 [*Halorhapdus tiamatea*], EAA28929.1 [*Neurospora crassa* OR74A], WP_011039146.1 [*Xanthomonas campestris*].
