# Supplementary material for: The structure of a GH149 β‐(1 → 3) glucan phosphorylase reveals a new surface oligosaccharide binding site and additional domains that are absent in the disaccharide‐specific GH94 glucose‐β‐(1 → 3)‐glucose (laminaribiose) phosphorylase
Source: Proteins. 2019 Jun 6;87(10):885–92. doi: 10.1002/prot.25745 (PMC6771811; doi:10.1002/prot.25745)
Supplement: Supplementary file 2 — Data S2 Supporting information [file PROT-87-885-s002.pdf]

Full wwPDB X-ray Structure Validation Report (i)

Sep 24, 2018 – 01:10 PM BST

PDB ID	:	6HQ6
Title	:	Bacterial beta-1,3-oligosaccharide phosphorylase from GH149
Deposited on	:	2018-09-24
Resolution	:	2.05 Å(reported)

This is a Full wwPDB X-ray Structure Validation Report.

This report is produced by the wwPDB biocuration pipeline after annotation of the structure.

We welcome your comments at *validation@mail.wwpdb.org*
A user guide is available at
https://www.wwpdb.org/validation/2017/XrayValidationReportHelp
with specific help available everywhere you see the (i) symbol.

The following versions of software and data (see references (i)) were used in the production of this report:

MolProbity	:	4.02b-467
Mogul	:	1.7.3 (157068), CSD as539be (2018)
Xtriage (Phenix)	:	1.13
EDS	:	rb-20031172
Percentile statistics	:	20171227.v01 (using entries in the PDB archive December 27th 2017)
Refmac	:	5.8.0158
CCP4	:	7.0 (Gargrove)
Ideal geometry (proteins)	:	Engh & Huber (2001)
Ideal geometry (DNA, RNA)	:	Parkinson et al. (1996)
Validation Pipeline (wwPDB-VP)	:	rb-20031172

CONFIDENTIAL VALIDATION REPORT

1 Overall quality at a glance (i)

The following experimental techniques were used to determine the structure:
X-RAY DIFFRACTION

The reported resolution of this entry is 2.05 Å.

Percentile scores (ranging between 0-100) for global validation metrics of the entry are shown in the following graphic. The table shows the number of entries on which the scores are based.

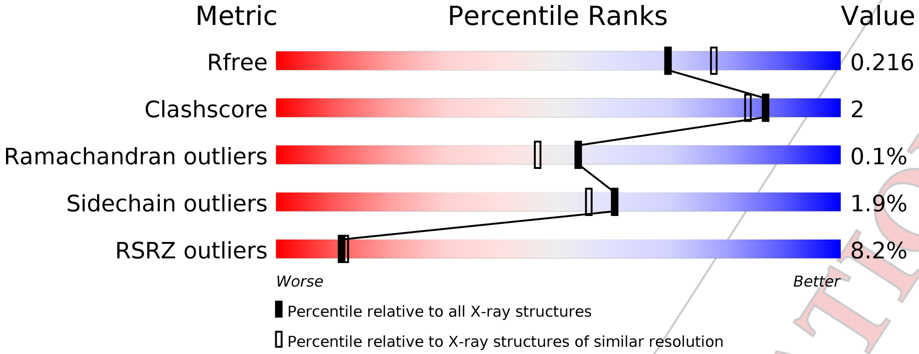

Metric	Whole archive (#Entries)	Similar resolution (#Entries, resolution range(Å))
R_{free}	111664	1449 (2.04-2.04)
Clashscore	122126	1524 (2.04-2.04)
Ramachandran outliers	120053	1512 (2.04-2.04)
Sidechain outliers	120020	1512 (2.04-2.04)
RSRZ outliers	108989	1429 (2.04-2.04)

The table below summarises the geometric issues observed across the polymeric chains and their fit to the electron density. The red, orange, yellow and green segments on the lower bar indicate the fraction of residues that contain outliers for >=3, 2, 1 and 0 types of geometric quality criteria. A grey segment represents the fraction of residues that are not modelled. The numeric value for each fraction is indicated below the corresponding segment, with a dot representing fractions <=5% The upper red bar (where present) indicates the fraction of residues that have poor fit to the electron density. The numeric value is given above the bar.

Mol	Chain	Length	Quality of chain
1	A	1175	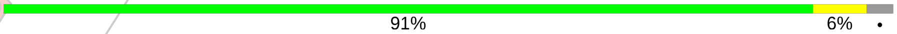
1	B	1175	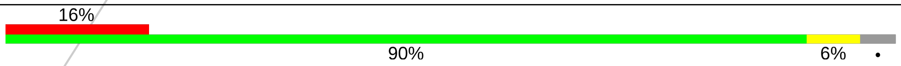

The following table lists non-polymeric compounds, carbohydrate monomers and non-standard residues in protein, DNA, RNA chains that are outliers for geometric or electron-density-fit criteria:

CONFIDENTIAL VALIDATION REPORT

Mol	Type	Chain	Res	Chirality	Geometry	Clashes	Electron density
4	EDO	A	1207	-	-	X	-
5	CL	A	1218[B]	-	-	X	-

CONFIDENTIAL VALIDATION REPORT

2 Entry composition (i)

There are 6 unique types of molecules in this entry. The entry contains 18854 atoms, of which 0 are hydrogens and 0 are deuteriums.

In the tables below, the ZeroOcc column contains the number of atoms modelled with zero occupancy, the AltConf column contains the number of residues with at least one atom in alternate conformation and the Trace column contains the number of residues modelled with at most 2 atoms.

- Molecule 1 is a protein called Bacterial beta-1,3-oligosaccharide phosphorylase.

Mol	Chain	Residues	Atoms					ZeroOcc	AltConf	Trace
1	A	1135	Total<br>9084	C<br>5805	N<br>1507	O<br>1743	S<br>29	0	3	0
1	B	1133	Total<br>8754	C<br>5578	N<br>1453	O<br>1694	S<br>29	0	0	0

- Molecule 2 is SULFATE ION (three-letter code: SO4) (formula: O_4S).

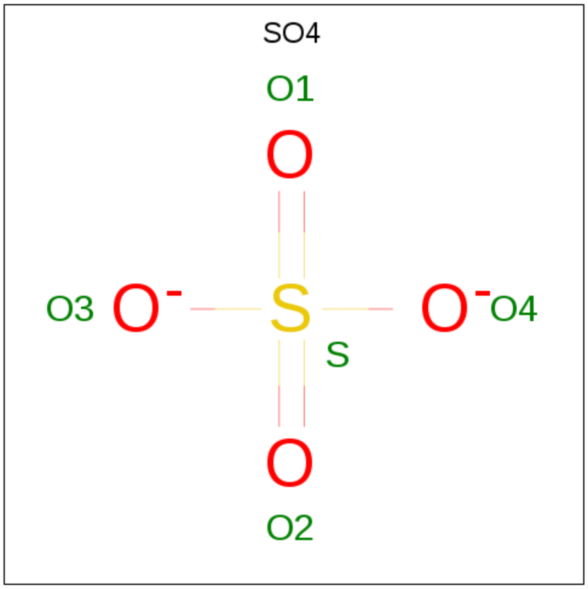

Mol	Chain	Residues	Atoms			ZeroOcc	AltConf
2	A	1	Total<br>5	O<br>4	S<br>1	0	0
2	A	1	Total<br>5	O<br>4	S<br>1	0	0
2	B	1	Total<br>5	O<br>4	S<br>1	0	0
2	B	1	Total<br>5	O<br>4	S<br>1	0	0

CONFIDENTIAL VALIDATION REPORT

- Molecule 3 is BICINE (three-letter code: BCN) (formula: $C_6H_{13}NO_4$).

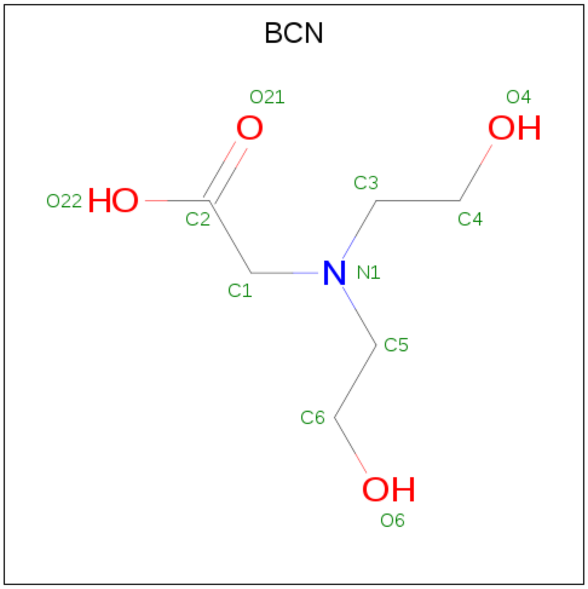

Mol	Chain	Residues	Atoms	ZeroOcc	AltConf
3	A	1	Total C N O<br>11 6 1 4	0	0
3	B	1	Total C N O<br>11 6 1 4	0	0

- Molecule 4 is 1,2-ETHANEDIOL (three-letter code: EDO) (formula: $C_2H_6O_2$).

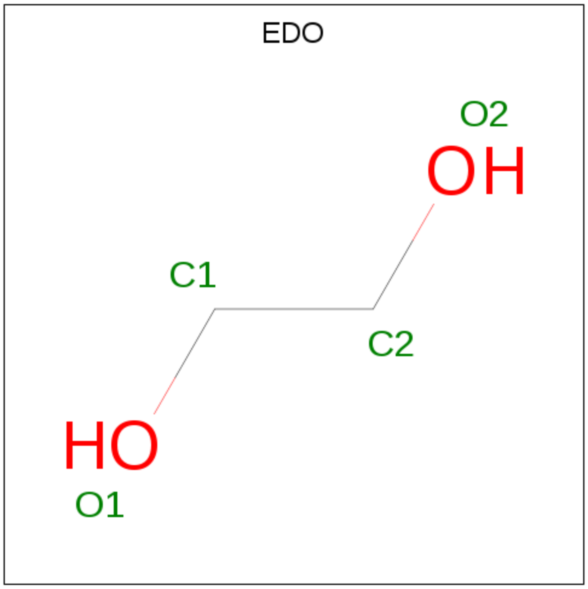

Mol	Chain	Residues	Atoms	ZeroOcc	AltConf
4	A	1	Total C O<br>4 2 2	0	0

Continued on next page...

CONFIDENTIAL VALIDATION REPORT

Continued from previous page...

Mol	Chain	Residues	Atoms	ZeroOcc	AltConf
4	A	1	Total C O<br>4 2 2	0	0
4	A	1	Total C O<br>4 2 2	0	0
4	A	1	Total C O<br>4 2 2	0	0
4	A	1	Total C O<br>4 2 2	0	0
4	A	1	Total C O<br>4 2 2	0	0
4	A	1	Total C O<br>4 2 2	0	0
4	A	1	Total C O<br>4 2 2	0	0
4	A	1	Total C O<br>4 2 2	0	0
4	A	1	Total C O<br>4 2 2	0	0
4	A	1	Total C O<br>4 2 2	0	0
4	A	1	Total C O<br>4 2 2	0	0
4	B	1	Total C O<br>4 2 2	0	0
4	B	1	Total C O<br>4 2 2	0	0
4	B	1	Total C O<br>4 2 2	0	0
4	B	1	Total C O<br>4 2 2	0	0
4	B	1	Total C O<br>4 2 2	0	0

- Molecule 5 is CHLORIDE ION (three-letter code: CL) (formula: Cl).

Mol	Chain	Residues	Atoms	ZeroOcc	AltConf
5	B	1	Total Cl<br>1 1	0	0
5	A	3	Total Cl<br>4 4	0	1

- Molecule 6 is water.

CONFIDENTIAL VALIDATION REPORT

Mol	Chain	Residues	Atoms		ZeroOcc	AltConf
6	A	676	Total<br>708	O<br>708	0	32
6	B	189	Total<br>193	O<br>193	0	4

CONFIDENTIAL VALIDATION REPORT

3 Residue-property plots ⓘ

These plots are drawn for all protein, RNA and DNA chains in the entry. The first graphic for a chain summarises the proportions of the various outlier classes displayed in the second graphic. The second graphic shows the sequence view annotated by issues in geometryand electron density. Residues are color-coded according to the number of geometric quality criteria for which they contain at least one outlier: green = 0, yellow = 1, orange = 2 and red = 3 or more. A red dot above a residue indicates a poor fit to the electron density (RSRZ > 2). Stretches of 2 or more consecutive residues without any outlier are shown as a green connector. Residues present in the sample, but not in the model, are shown in grey.

- Molecule 1: Bacterial beta-1,3-oligosaccharide phosphorylase

Chain A:

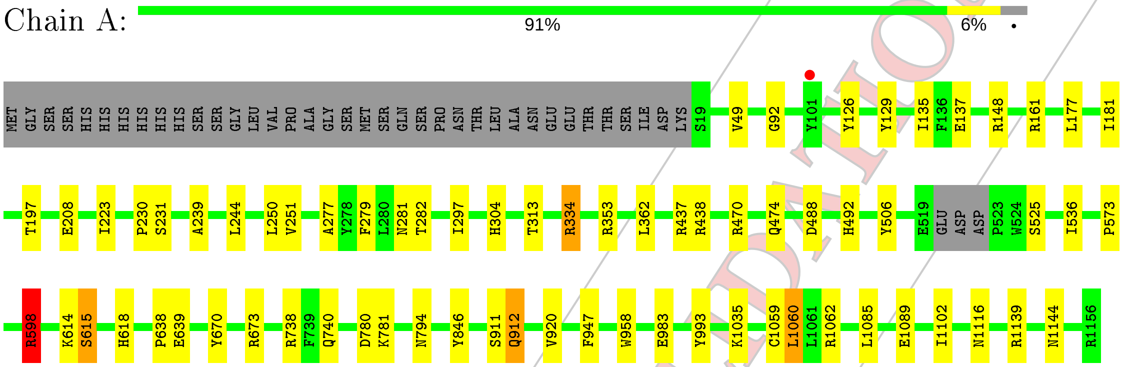

- Molecule 1: Bacterial beta-1,3-oligosaccharide phosphorylase

Chain B:

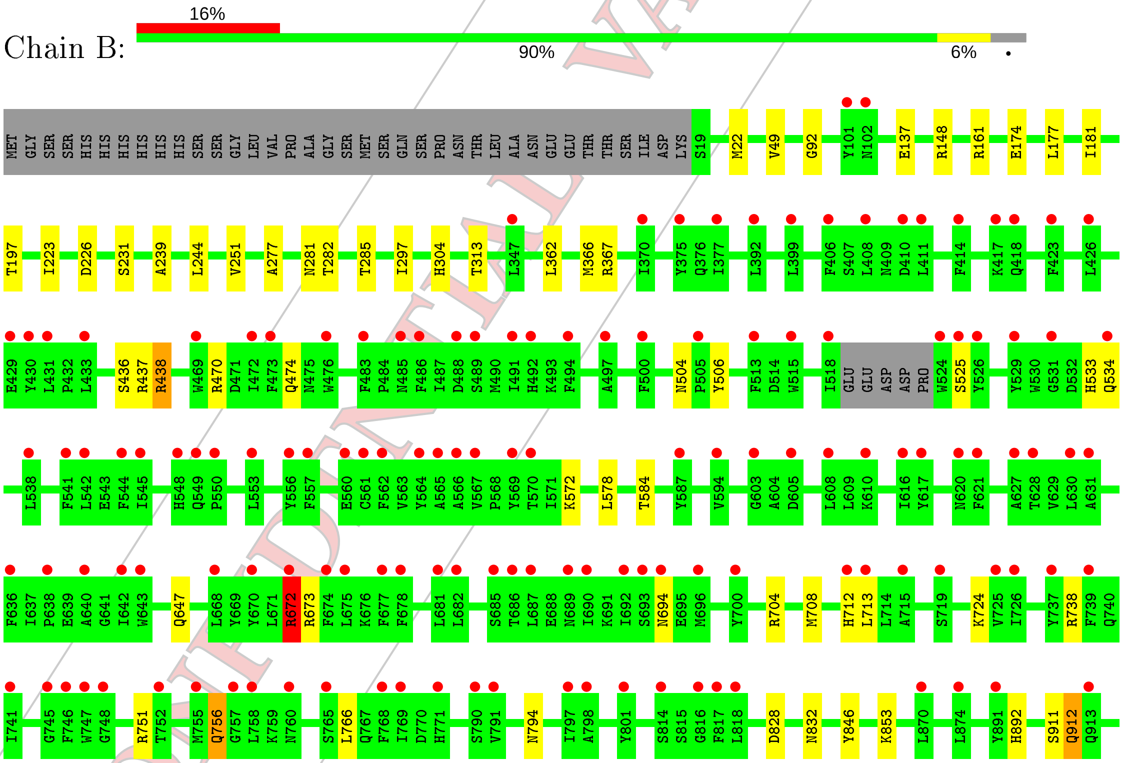

CONFIDENTIAL VALIDATION REPORT

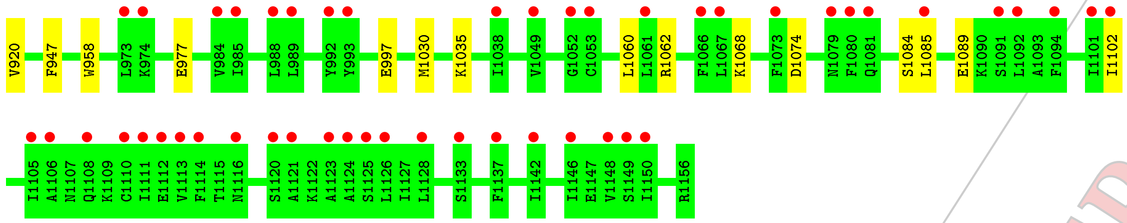

CONFIDENTIAL VALIDATION REPORT

4 Data and refinement statistics (i)

Property	Value	Source
Space group	P 21 21 21	Depositor
Cell constants<br>a, b, c, α, β, γ	100.24Å 158.99Å 181.56Å<br>90.00° 90.00° 90.00°	Depositor
Resolution (Å)	79.49 – 2.05<br>79.49 – 2.05	Depositor<br>EDS
% Data completeness<br>(in resolution range)	100.0 (79.49-2.05)<br>100.0 (79.49-2.05)	Depositor<br>EDS
R_{merge}	0.11	Depositor
R_{sym}	(Not available)	Depositor
$< I/\sigma(I) >$ [1]	1.47 (at 2.05Å)	Xtriage
Refinement program	REFMAC	Depositor
R, R_{free}	0.182 , 0.211<br>0.188 , 0.216	Depositor<br>DCC
R_{free} test set	9118 reflections (5.02%)	wwPDB-VP
Wilson B-factor (Å^2)	42.9	Xtriage
Anisotropy	0.229	Xtriage
Bulk solvent k_{sol}(e/Å^3), B_{sol}(Å^2)	0.32 , 61.2	EDS
L-test for twinning[2]	$< \|L\| >$ = 0.49, $< L^2 >$ = 0.32	Xtriage
Estimated twinning fraction	No twinning to report.	Xtriage
F_o,F_c correlation	0.96	EDS
Total number of atoms	18854	wwPDB-VP
Average B, all atoms (Å^2)	63.0	wwPDB-VP

Xtriage's analysis on translational NCS is as follows: *The largest off-origin peak in the Patterson function is 2.40% of the height of the origin peak. No significant pseudotranslation is detected.*

[1] Intensities estimated from amplitudes.

[2] Theoretical values of $< |L| >$, $< L^2 >$ for acentric reflections are 0.5, 0.333 respectively for untwinned datasets, and 0.375, 0.2 for perfectly twinned datasets.

CONFIDENTIAL VALIDATION REPORT

5 Model quality (i)

5.1 Standard geometry (i)

Bond lengths and bond angles in the following residue types are not validated in this section: BCN, CL, EDO, SO4

The Z score for a bond length (or angle) is the number of standard deviations the observed value is removed from the expected value. A bond length (or angle) with $|Z| > 5$ is considered an outlier worth inspection. RMSZ is the root-mean-square of all Z scores of the bond lengths (or angles).

Mol	Chain	Bond lengths		Bond angles	
		RMSZ	#$|Z|$ >5	RMSZ	#$|Z|$ >5
1	A	0.58	0/9284	0.68	3/12557 (0.0%)
1	B	0.46	0/8948	0.60	0/12154
All	All	0.53	0/18232	0.64	3/24711 (0.0%)

Chiral center outliers are detected by calculating the chiral volume of a chiral center and verifying if the center is modelled as a planar moiety or with the opposite hand.A planarity outlier is detected by checking planarity of atoms in a peptide group, atoms in a mainchain group or atoms of a sidechain that are expected to be planar.

Mol	Chain	#Chirality outliers	#Planarity outliers
1	A	0	6
1	B	0	4
All	All	0	10

There are no bond length outliers.

All (3) bond angle outliers are listed below:

Mol	Chain	Res	Type	Atoms	Z	Observed(°)	Ideal(°)
1	A	1139	ARG	NE-CZ-NH2	-8.65	115.97	120.30
1	A	598	ARG	NE-CZ-NH2	-5.18	117.71	120.30
1	A	334	ARG	NE-CZ-NH2	5.16	122.88	120.30

There are no chirality outliers.

All (10) planarity outliers are listed below:

Mol	Chain	Res	Type	Group
1	A	161	ARG	Sidechain
1	A	334	ARG	Sidechain
1	A	353	ARG	Sidechain

Continued on next page...

CONFIDENTIAL VALIDATION REPORT

Continued from previous page...

Mol	Chain	Res	Type	Group
1	A	438	ARG	Sidechain
1	A	598	ARG	Sidechain
1	A	673	ARG	Sidechain
1	B	1062	ARG	Sidechain
1	B	161	ARG	Sidechain
1	B	672	ARG	Sidechain
1	B	673	ARG	Sidechain

5.2 Too-close contacts (i)

In the following table, the Non-H and H(model) columns list the number of non-hydrogen atoms and hydrogen atoms in the chain respectively. The H(added) column lists the number of hydrogen atoms added and optimized by MolProbity. The Clashes column lists the number of clashes within the asymmetric unit, whereas Symm-Clashes lists symmetry related clashes.

Mol	Chain	Non-H	H(model)	H(added)	Clashes	Symm-Clashes
1	A	9084	0	8782	38	0
1	B	8754	0	8107	43	0
2	A	10	0	0	0	0
2	B	10	0	0	0	0
3	A	11	0	12	0	0
3	B	11	0	12	0	0
4	A	48	0	72	7	0
4	B	20	0	30	1	0
5	A	4	0	0	2	0
5	B	1	0	0	0	0
6	A	708	0	0	2	0
6	B	193	0	0	0	0
All	All	18854	0	17015	79	0

The all-atom clashscore is defined as the number of clashes found per 1000 atoms (including hydrogen atoms). The all-atom clashscore for this structure is 2.

All (79) close contacts within the same asymmetric unit are listed below, sorted by their clash magnitude.

Atom-1	Atom-2	Interatomic distance (Å)	Clash overlap (Å)
1:B:756:GLN:HA	1:B:756:GLN:HE21	1.29	0.96
1:B:438:ARG:HH11	1:B:438:ARG:HG2	1.30	0.93
1:A:912:GLN:HA	1:A:912:GLN:HE21	1.52	0.74
1:B:438:ARG:CG	1:B:438:ARG:HH11	2.01	0.74

Continued on next page...

CONFIDENTIAL VALIDATION REPORT

Continued from previous page...

Atom-1	Atom-2	Interatomic distance (Å)	Clash overlap (Å)
1:B:504:ASN:HD22	1:B:534:GLN:HE21	1.37	0.72
1:A:958:TRP:CG	4:A:1207:EDO:H21	2.28	0.69
1:B:504:ASN:HD21	1:B:533:HIS:HB3	1.59	0.66
1:B:912:GLN:HE21	1:B:912:GLN:HA	1.61	0.65
1:B:367:ARG:HB2	1:B:1030:MET:HE2	1.79	0.64
1:A:1116:ASN:HA	1:A:1144:ASN:HD21	1.63	0.64
1:B:438:ARG:NH1	1:B:438:ARG:CG	2.62	0.61
1:B:367:ARG:HA	1:B:1030:MET:HE3	1.83	0.60
1:B:367:ARG:CB	1:B:1030:MET:HE2	2.31	0.60
1:B:49:VAL:HG11	1:B:362:LEU:HD21	1.82	0.60
6:A:1909[B]:HOH:O	1:B:304:HIS:HD2	1.85	0.60
1:A:492:HIS:HD2	6:A:1634:HOH:O	1.89	0.55
1:A:993[B]:TYR:CE1	1:A:1062:ARG:NH2	2.74	0.54
1:B:438:ARG:NH1	1:B:438:ARG:HG2	2.10	0.54
1:B:756:GLN:NE2	1:B:756:GLN:HA	2.11	0.53
1:A:470:ARG:HG2	1:A:474:GLN:NE2	2.25	0.52
1:B:470:ARG:HG2	1:B:474:GLN:NE2	2.24	0.52
1:A:738:ARG:HG3	4:A:1205:EDO:H12	1.92	0.52
1:B:584:THR:H	1:B:647:GLN:NE2	2.08	0.51
1:A:598:ARG:NH2	1:A:615:SER:O	2.42	0.50
1:B:672:ARG:HH21	1:B:766:LEU:HD22	1.76	0.49
1:B:244:LEU:HD22	1:B:282:THR:HG21	1.95	0.49
1:A:304:HIS:CE1	1:B:892:HIS:CE1	3.01	0.49
1:A:49:VAL:HG21	1:A:362:LEU:HD21	1.95	0.49
1:B:712:HIS:CE1	1:B:713:LEU:HD12	2.47	0.48
1:B:756:GLN:CA	1:B:756:GLN:HE21	2.10	0.48
1:A:573:PRO:HG3	4:A:1205:EDO:H22	1.96	0.47
1:B:704:ARG:HG2	1:B:708:MET:SD	2.54	0.47
1:B:578:LEU:HD21	1:B:724:LYS:NZ	2.29	0.47
1:B:912:GLN:CA	1:B:912:GLN:HE21	2.25	0.47
1:B:958:TRP:CD1	4:B:1206:EDO:H21	2.50	0.47
1:B:174:GLU:OE1	1:B:285:THR:OG1	2.26	0.47
1:B:1074:ASP:OD1	1:B:1084:SER:OG	2.32	0.47
1:A:912:GLN:NE2	1:A:912:GLN:HA	2.25	0.46
1:B:223:ILE:HB	1:B:231:SER:OG	2.16	0.46
1:B:367:ARG:HA	1:B:1030:MET:CE	2.45	0.46
1:A:1085:LEU:HD13	1:A:1102:ILE:HD11	1.97	0.46
1:B:694:ASN:HD22	1:B:751:ARG:CG	2.28	0.46
1:A:137:GLU:OE2	1:A:148:ARG:HD3	2.16	0.45
1:B:572:LYS:O	1:B:738:ARG:NH2	2.49	0.45
1:B:137:GLU:OE2	1:B:148:ARG:HD3	2.16	0.45

Continued on next page...

CONFIDENTIAL VALIDATION REPORT

Continued from previous page...

Atom-1	Atom-2	Interatomic distance (Å)	Clash overlap (Å)
1:A:958:TRP:CD1	4:A:1207:EDO:H21	2.52	0.45
1:B:1085:LEU:HD13	1:B:1102:ILE:HD11	1.99	0.45
1:B:828:ASP:O	1:B:832:ASN:ND2	2.50	0.45
1:A:1116:ASN:HA	1:A:1144:ASN:ND2	2.32	0.44
1:A:911:SER:HB3	1:A:920:VAL:HG11	2.00	0.44
1:A:958:TRP:CD1	4:A:1207:EDO:C2	3.01	0.44
1:A:223:ILE:HB	1:A:231:SER:OG	2.18	0.44
1:A:49:VAL:HG21	1:A:362:LEU:CD2	2.48	0.44
1:B:177:LEU:O	1:B:281:ASN:HA	2.17	0.44
1:A:244:LEU:HD22	1:A:282:THR:HG21	2.00	0.44
1:B:911:SER:HB3	1:B:920:VAL:HG11	1.99	0.44
1:A:251:VAL:HA	1:A:277:ALA:O	2.18	0.43
1:B:367:ARG:CA	1:B:1030:MET:HE3	2.47	0.43
1:A:618:HIS:ND1	5:A:1218[B]:CL:CL	2.77	0.43
1:A:92:GLY:O	1:A:181:ILE:HA	2.18	0.43
1:B:92:GLY:O	1:B:181:ILE:HA	2.19	0.42
1:A:177:LEU:O	1:A:281:ASN:HA	2.18	0.42
1:A:129:TYR:HB3	4:A:1214:EDO:C2	2.50	0.42
1:A:780:ASP:O	1:A:781:LYS:HB2	2.20	0.41
1:A:993[B]:TYR:CE1	1:A:1062:ARG:CZ	3.03	0.41
1:B:366:MET:O	1:B:436:SER:HA	2.20	0.41
1:A:1059:CYS:SG	1:A:1060:LEU:HD13	2.61	0.41
1:A:614:LYS:HG3	5:A:1218[B]:CL:CL	2.58	0.41
1:A:250:LEU:HB2	1:A:279:PHE:HB2	2.02	0.41
1:A:536:ILE:HD13	1:A:670:TYR:CD1	2.55	0.41
1:B:239:ALA:HA	1:B:297:ILE:O	2.20	0.41
1:B:251:VAL:HA	1:B:277:ALA:O	2.20	0.41
1:A:958:TRP:CG	4:A:1207:EDO:C2	3.03	0.40
1:A:208:GLU:OE1	1:B:853:LYS:HB3	2.22	0.40
1:A:638:PRO:O	1:A:639:GLU:HB2	2.22	0.40
1:A:239:ALA:HA	1:A:297:ILE:O	2.21	0.40
1:A:126:TYR:HB2	1:A:135:ILE:HB	2.04	0.40
1:A:177:LEU:C	1:A:177:LEU:HD12	2.42	0.40
1:B:177:LEU:C	1:B:177:LEU:HD12	2.42	0.40

There are no symmetry-related clashes.

CONFIDENTIAL VALIDATION REPORT

5.3 Torsion angles (i)

5.3.1 Protein backbone (i)

In the following table, the Percentiles column shows the percent Ramachandran outliers of the chain as a percentile score with respect to all X-ray entries followed by that with respect to entries of similar resolution.

The Analysed column shows the number of residues for which the backbone conformation was analysed, and the total number of residues.

Mol	Chain	Analysed	Favoured	Allowed	Outliers	Percentiles	
1	A	1134/1175 (96%)	1102 (97%)	31 (3%)	1 (0%)	53	46
1	B	1129/1175 (96%)	1095 (97%)	33 (3%)	1 (0%)	53	46
All	All	2263/2350 (96%)	2197 (97%)	64 (3%)	2 (0%)	53	46

All (2) Ramachandran outliers are listed below:

Mol	Chain	Res	Type
1	A	794	ASN
1	B	794	ASN

5.3.2 Protein sidechains (i)

In the following table, the Percentiles column shows the percent sidechain outliers of the chain as a percentile score with respect to all X-ray entries followed by that with respect to entries of similar resolution.

The Analysed column shows the number of residues for which the sidechain conformation was analysed, and the total number of residues.

Mol	Chain	Analysed	Rotameric	Outliers	Percentiles	
1	A	979/1041 (94%)	962 (98%)	17 (2%)	63	60
1	B	892/1041 (86%)	873 (98%)	19 (2%)	56	51
All	All	1871/2082 (90%)	1835 (98%)	36 (2%)	60	55

All (36) residues with a non-rotameric sidechain are listed below:

Mol	Chain	Res	Type
1	A	197	THR
1	A	230	PRO
1	A	313	THR
1	A	437	ARG

Continued on next page...

CONFIDENTIAL VALIDATION REPORT

Continued from previous page...

Mol	Chain	Res	Type
1	A	488[A]	ASP
1	A	488[B]	ASP
1	A	506	TYR
1	A	525	SER
1	A	615	SER
1	A	740	GLN
1	A	846	TYR
1	A	912	GLN
1	A	947	PHE
1	A	983	GLU
1	A	1035	LYS
1	A	1060	LEU
1	A	1089	GLU
1	B	22	MET
1	B	197	THR
1	B	226	ASP
1	B	313	THR
1	B	437	ARG
1	B	438	ARG
1	B	506	TYR
1	B	525	SER
1	B	672	ARG
1	B	756	GLN
1	B	846	TYR
1	B	912	GLN
1	B	947	PHE
1	B	977	GLU
1	B	997	GLU
1	B	1035	LYS
1	B	1060	LEU
1	B	1068	LYS
1	B	1089	GLU

Some sidechains can be flipped to improve hydrogen bonding and reduce clashes. All (16) such sidechains are listed below:

Mol	Chain	Res	Type
1	A	100	GLN
1	A	156	GLN
1	A	194	GLN
1	A	680	GLN
1	A	684	ASN
1	A	912	GLN

Continued on next page...

CONFIDENTIAL VALIDATION REPORT

Continued from previous page...

Mol	Chain	Res	Type
1	A	1144	ASN
1	B	100	GLN
1	B	156	GLN
1	B	504	ASN
1	B	647	GLN
1	B	684	ASN
1	B	694	ASN
1	B	712	HIS
1	B	756	GLN
1	B	912	GLN

5.3.3 RNA (i)

There are no RNA molecules in this entry.

5.4 Non-standard residues in protein, DNA, RNA chains (i)

There are no non-standard protein/DNA/RNA residues in this entry.

5.5 Carbohydrates (i)

There are no carbohydrates in this entry.

5.6 Ligand geometry (i)

Of 28 ligands modelled in this entry, 5 are monoatomic - leaving 23 for Mogul analysis.

In the following table, the Counts columns list the number of bonds (or angles) for which Mogul statistics could be retrieved, the number of bonds (or angles) that are observed in the model and the number of bonds (or angles) that are defined in the Chemical Component Dictionary. The Link column lists molecule types, if any, to which the group is linked. The Z score for a bond length (or angle) is the number of standard deviations the observed value is removed from the expected value. A bond length (or angle) with $|Z| > 2$ is considered an outlier worth inspection. RMSZ is the root-mean-square of all Z scores of the bond lengths (or angles).

Mol	Type	Chain	Res	Link	Bond lengths			Bond angles		
					Counts	RMSZ	$\#\|Z\| > 2$	Counts	RMSZ	$\#\|Z\| > 2$
2	SO4	A	1201	-	4,4,4	0.66	0	6,6,6	0.71	0
2	SO4	A	1202	-	4,4,4	0.24	0	6,6,6	0.81	0
3	BCN	A	1203	-	7,10,10	0.45	0	8,11,11	1.30	1 (12%)
4	EDO	A	1204	-	3,3,3	0.50	0	2,2,2	0.36	0

CONFIDENTIAL VALIDATION REPORT

CONFIDENTIAL VALIDATION REPORT

Mol	Type	Chain	Res	Link	Bond lengths			Bond angles		
					Counts	RMSZ	#|Z| > 2	Counts	RMSZ	#|Z| > 2
4	EDO	A	1205	-	3,3,3	0.28	0	2,2,2	0.72	0
4	EDO	A	1206	-	3,3,3	0.36	0	2,2,2	0.33	0
4	EDO	A	1207	-	3,3,3	0.56	0	2,2,2	0.17	0
4	EDO	A	1208	-	3,3,3	0.53	0	2,2,2	0.12	0
4	EDO	A	1209	-	3,3,3	0.42	0	2,2,2	0.52	0
4	EDO	A	1210	-	3,3,3	0.41	0	2,2,2	0.82	0
4	EDO	A	1211	-	3,3,3	0.49	0	2,2,2	0.54	0
4	EDO	A	1212	-	3,3,3	0.54	0	2,2,2	0.37	0
4	EDO	A	1213	-	3,3,3	0.57	0	2,2,2	0.34	0
4	EDO	A	1214	-	3,3,3	0.40	0	2,2,2	0.24	0
4	EDO	A	1215	-	3,3,3	0.43	0	2,2,2	0.46	0
2	SO4	B	1201	-	4,4,4	0.46	0	6,6,6	0.33	0
2	SO4	B	1202	-	4,4,4	0.42	0	6,6,6	0.25	0
3	BCN	B	1203	-	7,10,10	0.54	0	8,11,11	1.26	1 (12%)
4	EDO	B	1204	-	3,3,3	0.57	0	2,2,2	0.21	0
4	EDO	B	1205	-	3,3,3	0.56	0	2,2,2	0.18	0
4	EDO	B	1206	-	3,3,3	0.63	0	2,2,2	0.09	0
4	EDO	B	1207	-	3,3,3	0.48	0	2,2,2	0.35	0
4	EDO	B	1208	-	3,3,3	0.31	0	2,2,2	0.58	0

In the following table, the Chirals column lists the number of chiral outliers, the number of chiral centers analysed, the number of these observed in the model and the number defined in the Chemical Component Dictionary. Similar counts are reported in the Torsion and Rings columns. '-' means no outliers of that kind were identified.

Mol	Type	Chain	Res	Link	Chirals	Torsions	Rings
2	SO4	A	1201	-	-	0/0/0/0	0/0/0/0
2	SO4	A	1202	-	-	0/0/0/0	0/0/0/0
3	BCN	A	1203	-	-	0/8/10/10	0/0/0/0
4	EDO	A	1204	-	-	0/1/1/1	0/0/0/0
4	EDO	A	1205	-	-	0/1/1/1	0/0/0/0
4	EDO	A	1206	-	-	0/1/1/1	0/0/0/0
4	EDO	A	1207	-	-	0/1/1/1	0/0/0/0
4	EDO	A	1208	-	-	0/1/1/1	0/0/0/0
4	EDO	A	1209	-	-	0/1/1/1	0/0/0/0
4	EDO	A	1210	-	-	0/1/1/1	0/0/0/0
4	EDO	A	1211	-	-	0/1/1/1	0/0/0/0
4	EDO	A	1212	-	-	0/1/1/1	0/0/0/0
4	EDO	A	1213	-	-	0/1/1/1	0/0/0/0
4	EDO	A	1214	-	-	0/1/1/1	0/0/0/0
4	EDO	A	1215	-	-	0/1/1/1	0/0/0/0
2	SO4	B	1201	-	-	0/0/0/0	0/0/0/0

Continued on next page...

Continued from previous page...

Mol	Type	Chain	Res	Link	Chirals	Torsions	Rings
2	SO4	B	1202	-	-	0/0/0/0	0/0/0/0
3	BCN	B	1203	-	-	0/8/10/10	0/0/0/0
4	EDO	B	1204	-	-	0/1/1/1	0/0/0/0
4	EDO	B	1205	-	-	0/1/1/1	0/0/0/0
4	EDO	B	1206	-	-	0/1/1/1	0/0/0/0
4	EDO	B	1207	-	-	0/1/1/1	0/0/0/0
4	EDO	B	1208	-	-	0/1/1/1	0/0/0/0

There are no bond length outliers.

All (2) bond angle outliers are listed below:

Mol	Chain	Res	Type	Atoms	Z	Observed(^{o})	Ideal(^{o})
3	A	1203	BCN	C2-C1-N1	-3.03	109.17	113.48
3	B	1203	BCN	C2-C1-N1	-2.79	109.50	113.48

There are no chirality outliers.

There are no torsion outliers.

There are no ring outliers.

4 monomers are involved in 8 short contacts:

Mol	Chain	Res	Type	Clashes	Symm-Clashes
4	A	1205	EDO	2	0
4	A	1207	EDO	4	0
4	A	1214	EDO	1	0
4	B	1206	EDO	1	0

5.7 Other polymers (i)

There are no such residues in this entry.

5.8 Polymer linkage issues (i)

There are no chain breaks in this entry.

CONFIDENTIAL VALIDATION REPORT

6 Fit of model and data (i)

6.1 Protein, DNA and RNA chains (i)

In the following table, the column labelled '#RSRZ> 2' contains the number (and percentage) of RSRZ outliers, followed by percent RSRZ outliers for the chain as percentile scores relative to all X-ray entries and entries of similar resolution. The OWAB column contains the minimum, median, 95th percentile and maximum values of the occupancy-weighted average B-factor per residue. The column labelled 'Q< 0.9' lists the number of (and percentage) of residues with an average occupancy less than 0.9.

Mol	Chain	Analysed	<RSRZ>	#RSRZ>2			OWAB(Å^2)	Q<0.9
1	A	1135/1175 (96%)	-0.10	1 (0%)	95	95	29, 44, 75, 108	0
1	B	1133/1175 (96%)	0.74	186 (16%)	1	1	36, 82, 124, 145	0
All	All	2268/2350 (96%)	0.32	187 (8%)	11	12	29, 58, 116, 145	0

All (187) RSRZ outliers are listed below:

Mol	Chain	Res	Type	RSRZ
1	B	423	PHE	8.5
1	B	1128	LEU	6.4
1	B	486	PHE	6.0
1	B	524	TRP	5.8
1	B	681	LEU	5.8
1	B	814	SER	5.6
1	B	817	PHE	5.5
1	B	758	LEU	5.4
1	B	541	PHE	5.2
1	B	1111	ILE	5.0
1	B	1113	VAL	4.8
1	B	525	SER	4.7
1	B	640	ALA	4.6
1	B	674	PHE	4.5
1	B	538	LEU	4.4
1	B	542	LEU	4.4
1	B	696	MET	4.4
1	B	1123	ALA	4.4
1	B	816	GLY	4.3
1	B	392	LEU	4.3
1	B	643	TRP	4.3
1	B	801	TYR	4.3
1	B	411	LEU	4.3
1	B	548	HIS	4.3

Continued on next page...

CONFIDENTIAL VALIDATION REPORT

Continued from previous page...

Mol	Chain	Res	Type	RSRZ
1	B	489	SER	4.2
1	B	545	ILE	4.2
1	B	500	PHE	4.2
1	B	973	LEU	4.2
1	B	1105	ILE	4.1
1	B	406	PHE	4.1
1	B	747	TRP	4.0
1	B	565	ALA	3.9
1	B	748	GLY	3.9
1	B	687	LEU	3.9
1	B	1121	ALA	3.9
1	B	569	TYR	3.9
1	B	1114	PHE	3.9
1	B	531	GLY	3.9
1	B	746	PHE	3.8
1	B	560	GLU	3.8
1	B	608	LEU	3.8
1	B	497	ALA	3.8
1	B	985	ILE	3.8
1	B	408	LEU	3.8
1	B	1125	SER	3.7
1	B	553	LEU	3.7
1	B	675	LEU	3.7
1	B	1148	VAL	3.7
1	B	1061	LEU	3.7
1	B	870	LEU	3.6
1	B	566	ALA	3.6
1	B	431	LEU	3.6
1	B	984	VAL	3.6
1	B	1124	ALA	3.5
1	B	636	PHE	3.5
1	B	526	TYR	3.5
1	B	752	THR	3.4
1	B	557	PHE	3.4
1	B	621	PHE	3.4
1	B	562	PHE	3.3
1	B	1110	CYS	3.3
1	B	375	TYR	3.3
1	B	377	ILE	3.3
1	B	594	VAL	3.3
1	B	678	PHE	3.3
1	B	426	LEU	3.3

Continued on next page...

CONFIDENTIAL VALIDATION REPORT

Continued from previous page...

Mol	Chain	Res	Type	RSRZ
1	B	682	LEU	3.3
1	B	769	ILE	3.2
1	B	1120	SER	3.2
1	B	993	TYR	3.2
1	B	715	ALA	3.2
1	B	567	VAL	3.2
1	B	556	TYR	3.1
1	B	797	ILE	3.1
1	B	677	PHE	3.1
1	B	1142	ILE	3.1
1	B	429	GLU	3.0
1	B	668	LEU	3.0
1	A	101	TYR	3.0
1	B	414	PHE	2.9
1	B	494	PHE	2.9
1	B	544	PHE	2.9
1	B	1038	ILE	2.9
1	B	1116	ASN	2.9
1	B	1102	ILE	2.9
1	B	1150	ILE	2.9
1	B	485	ASN	2.9
1	B	686	THR	2.9
1	B	628	THR	2.8
1	B	719	SER	2.8
1	B	472	ILE	2.8
1	B	798	ALA	2.8
1	B	1101	ILE	2.8
1	B	689	ASN	2.8
1	B	1052	GLY	2.8
1	B	790	SER	2.8
1	B	791	VAL	2.7
1	B	642	ILE	2.7
1	B	101	TYR	2.7
1	B	1049	VAL	2.7
1	B	1146	ILE	2.7
1	B	430	TYR	2.7
1	B	1053	CYS	2.7
1	B	1079	ASN	2.7
1	B	564	TYR	2.7
1	B	891	TYR	2.7
1	B	1091	SER	2.6
1	B	347	LEU	2.6

Continued on next page...

CONFIDENTIAL VALIDATION REPORT

Continued from previous page...

Mol	Chain	Res	Type	RSRZ
1	B	818	LEU	2.6
1	B	617	TYR	2.6
1	B	483	PHE	2.6
1	B	1112	GLU	2.6
1	B	765	SER	2.6
1	B	476	TRP	2.6
1	B	513	PHE	2.5
1	B	587	TYR	2.5
1	B	700	TYR	2.5
1	B	550	PRO	2.5
1	B	1080	PHE	2.5
1	B	1092	LEU	2.5
1	B	561	CYS	2.5
1	B	712	HIS	2.5
1	B	739	PHE	2.5
1	B	1133	SER	2.5
1	B	1149	SER	2.5
1	B	473	PHE	2.5
1	B	631	ALA	2.5
1	B	638	PRO	2.5
1	B	410	ASP	2.5
1	B	992	TYR	2.5
1	B	745	GLY	2.5
1	B	492	HIS	2.4
1	B	102	ASN	2.4
1	B	1108	GLN	2.4
1	B	741	ILE	2.4
1	B	1066	PHE	2.4
1	B	1137	PHE	2.4
1	B	989	LEU	2.4
1	B	603	GLY	2.4
1	B	757	GLY	2.3
1	B	529	TYR	2.3
1	B	737	TYR	2.3
1	B	974	LYS	2.3
1	B	570	THR	2.3
1	B	370	ILE	2.3
1	B	418	GLN	2.3
1	B	690	ILE	2.3
1	B	988	LEU	2.3
1	B	1081	GLN	2.3
1	B	491	ILE	2.3

Continued on next page...

CONFIDENTIAL VALIDATION REPORT

Continued from previous page...

Mol	Chain	Res	Type	RSRZ
1	B	771	HIS	2.3
1	B	534	GLN	2.3
1	B	399	LEU	2.2
1	B	874	LEU	2.2
1	B	1073	PHE	2.2
1	B	627	ALA	2.2
1	B	1067	LEU	2.2
1	B	1106	ALA	2.2
1	B	913	GLN	2.2
1	B	694	ASN	2.2
1	B	469	TRP	2.2
1	B	693	SER	2.2
1	B	670	TYR	2.2
1	B	488	ASP	2.2
1	B	685	SER	2.2
1	B	433	LEU	2.1
1	B	518	ILE	2.1
1	B	725	VAL	2.1
1	B	630	LEU	2.1
1	B	505	PRO	2.1
1	B	760	ASN	2.1
1	B	616	ILE	2.1
1	B	1085	LEU	2.1
1	B	610	LYS	2.1
1	B	755	MET	2.1
1	B	1094	PHE	2.1
1	B	692	ILE	2.1
1	B	713	LEU	2.1
1	B	1126	LEU	2.1
1	B	672	ARG	2.1
1	B	768	PHE	2.1
1	B	620	ASN	2.0
1	B	726	ILE	2.0
1	B	417	LYS	2.0
1	B	605	ASP	2.0
1	B	549	GLN	2.0
1	B	515	TRP	2.0

6.2 Non-standard residues in protein, DNA, RNA chains ⓘ

There are no non-standard protein/DNA/RNA residues in this entry.

CONFIDENTIAL VALIDATION REPORT

6.3 Carbohydrates (i)

There are no carbohydrates in this entry.

6.4 Ligands (i)

In the following table, the Atoms column lists the number of modelled atoms in the group and the number defined in the chemical component dictionary. The B-factors column lists the minimum, median, 95th percentile and maximum values of B factors of atoms in the group. The column labelled 'Q< 0.9' lists the number of atoms with occupancy less than 0.9.

Mol	Type	Chain	Res	Atoms	RSCC	RSR	B-factors($Å^2$)	Q<0.9
2	SO4	B	1202	5/5	0.72	0.13	116,122,130,137	0
3	BCN	B	1203	11/11	0.79	0.16	66,71,80,80	0
4	EDO	A	1211	4/4	0.84	0.14	47,57,59,65	0
5	CL	A	1217	1/1	0.86	0.14	103,103,103,103	0
4	EDO	B	1207	4/4	0.87	0.19	67,73,74,76	0
5	CL	B	1209	1/1	0.88	0.16	92,92,92,92	0
5	CL	A	1218[A]	1/1	0.89	0.26	48,48,48,48	1
5	CL	A	1218[B]	1/1	0.89	0.26	67,67,67,67	1
4	EDO	A	1215	4/4	0.89	0.23	65,68,71,74	0
4	EDO	B	1205	4/4	0.90	0.12	71,75,78,78	0
4	EDO	B	1206	4/4	0.90	0.23	64,66,71,72	0
4	EDO	A	1213	4/4	0.91	0.11	57,57,60,61	0
4	EDO	A	1208	4/4	0.92	0.10	44,44,46,48	0
4	EDO	B	1204	4/4	0.92	0.11	55,60,60,63	0
5	CL	A	1216	1/1	0.92	0.17	78,78,78,78	0
4	EDO	A	1207	4/4	0.93	0.12	37,42,44,44	0
4	EDO	A	1209	4/4	0.93	0.14	42,47,52,58	0
3	BCN	A	1203	11/11	0.94	0.12	34,39,45,46	0
4	EDO	A	1206	4/4	0.94	0.13	52,52,57,57	0
4	EDO	A	1212	4/4	0.94	0.16	57,60,65,66	0
2	SO4	B	1201	5/5	0.95	0.14	77,80,83,95	0
4	EDO	A	1210	4/4	0.96	0.15	33,35,36,42	0
4	EDO	A	1214	4/4	0.97	0.17	51,56,62,64	0
4	EDO	B	1208	4/4	0.97	0.13	59,60,61,66	0
4	EDO	A	1205	4/4	0.97	0.24	45,45,46,47	0
4	EDO	A	1204	4/4	0.97	0.13	35,36,36,37	0
2	SO4	A	1201	5/5	0.98	0.09	48,50,54,56	0
2	SO4	A	1202	5/5	0.99	0.14	48,50,51,62	0

6.5 Other polymers (i)

There are no such residues in this entry.

CONFIDENTIAL VALIDATION REPORT
