# Supplementary material for: The structure of a GH149 β‐(1 → 3) glucan phosphorylase reveals a new surface oligosaccharide binding site and additional domains that are absent in the disaccharide‐specific GH94 glucose‐β‐(1 → 3)‐glucose (laminaribiose) phosphorylase
Source: Proteins. 2019 Jun 6;87(10):885–92. doi: 10.1002/prot.25745 (PMC6771811; doi:10.1002/prot.25745)
Supplement: Supplementary file 3 — Data S3 Supporting information [file PROT-87-885-s003.pdf]

Full wwPDB X-ray Structure Validation Report (i)

Sep 24, 2018 – 02:43 PM BST

PDB ID	:	6HQ8
Title	:	Bacterial beta-1,3-oligosaccharide phosphorylase from GH149 with laminarihexaose bound at a surface site
Deposited on	:	2018-09-24
Resolution	:	2.25 Å(reported)

This is a Full wwPDB X-ray Structure Validation Report.

This report is produced by the wwPDB biocuration pipeline after annotation of the structure.

We welcome your comments at *validation@mail.wwpdb.org*
A user guide is available at
https://www.wwpdb.org/validation/2017/XrayValidationReportHelp
with specific help available everywhere you see the (i) symbol.

CONFIDENTIAL VALIDATION REPORT

The following versions of software and data (see references (i)) were used in the production of this report:

MolProbity	:	4.02b-467
Mogul	:	1.7.3 (157068), CSD as539be (2018)
Xtriage (Phenix)	:	1.13
EDS	:	rb-20031172
Percentile statistics	:	20171227.v01 (using entries in the PDB archive December 27th 2017)
Refmac	:	5.8.0158
CCP4	:	7.0 (Gargrove)
Ideal geometry (proteins)	:	Engh & Huber (2001)
Ideal geometry (DNA, RNA)	:	Parkinson et al. (1996)
Validation Pipeline (wwPDB-VP)	:	rb-20031172

1 Overall quality at a glance (i)

The following experimental techniques were used to determine the structure:
X-RAY DIFFRACTION

The reported resolution of this entry is 2.25 Å.

Percentile scores (ranging between 0-100) for global validation metrics of the entry are shown in the following graphic. The table shows the number of entries on which the scores are based.

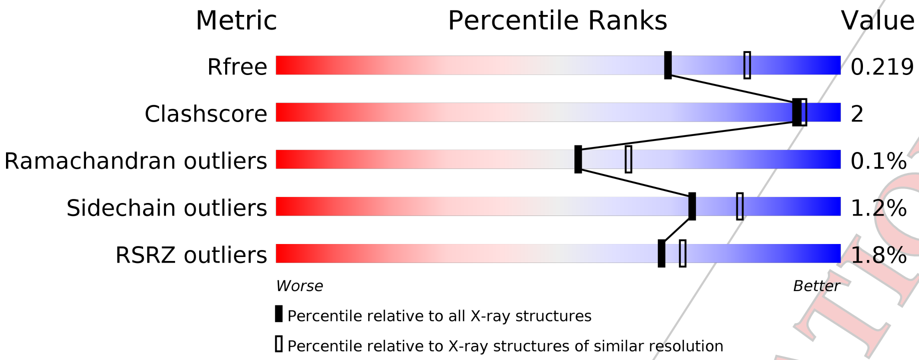

Metric	Whole archive (#Entries)	Similar resolution (#Entries, resolution range(Å))
R_{free}	111664	1178 (2.26-2.26)
Clashscore	122126	1286 (2.26-2.26)
Ramachandran outliers	120053	1253 (2.26-2.26)
Sidechain outliers	120020	1254 (2.26-2.26)
RSRZ outliers	108989	1158 (2.26-2.26)

The table below summarises the geometric issues observed across the polymeric chains and their fit to the electron density. The red, orange, yellow and green segments on the lower bar indicate the fraction of residues that contain outliers for >=3, 2, 1 and 0 types of geometric quality criteria. A grey segment represents the fraction of residues that are not modelled. The numeric value for each fraction is indicated below the corresponding segment, with a dot representing fractions <=5% The upper red bar (where present) indicates the fraction of residues that have poor fit to the electron density. The numeric value is given above the bar.

Mol	Chain	Length	Quality of chain
1	A	1175	92% 5% •
1	B	1175	3% 93% • •

CONFIDENTIAL VALIDATION REPORT

2 Entry composition (i)

There are 7 unique types of molecules in this entry. The entry contains 18945 atoms, of which 0 are hydrogens and 0 are deuteriums.

In the tables below, the ZeroOcc column contains the number of atoms modelled with zero occupancy, the AltConf column contains the number of residues with at least one atom in alternate conformation and the Trace column contains the number of residues modelled with at most 2 atoms.

- Molecule 1 is a protein called Beta-1,3-oligosaccharide phosphorylase.

Mol	Chain	Residues	Atoms					ZeroOcc	AltConf	Trace
			Total	C	N	O	S			
1	A	1138	9067	5789	1507	1742	29	0	1	0
1	B	1138	8926	5697	1479	1721	29	0	1	0

- Molecule 2 is SULFATE ION (three-letter code: SO4) (formula: O_4S).

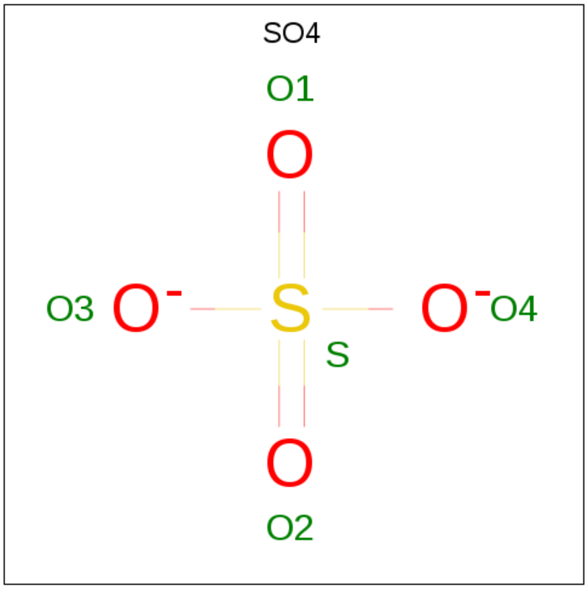

Mol	Chain	Residues	Atoms			ZeroOcc	AltConf
			Total	O	S		
2	A	1	5	4	1	0	0
2	A	1	5	4	1	0	0
2	B	1	5	4	1	0	0
2	B	1	5	4	1	0	0

CONFIDENTIAL VALIDATION REPORT

- Molecule 3 is BICINE (three-letter code: BCN) (formula: $C_6H_{13}NO_4$).

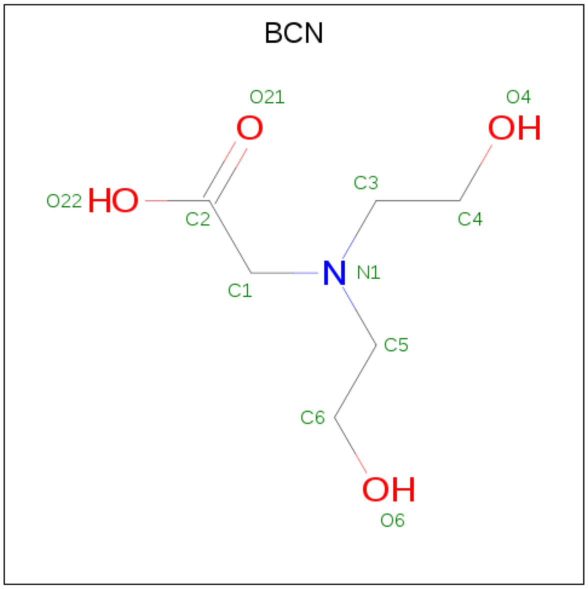

Mol	Chain	Residues	Atoms	ZeroOcc	AltConf
3	A	1	Total C N O<br>11 6 1 4	0	0
3	B	1	Total C N O<br>11 6 1 4	0	0

- Molecule 4 is 1,2-ETHANEDIOL (three-letter code: EDO) (formula: $C_2H_6O_2$).

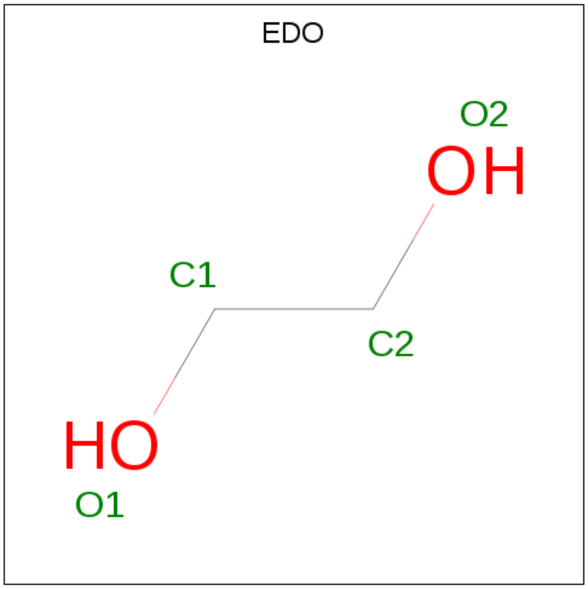

Mol	Chain	Residues	Atoms	ZeroOcc	AltConf
4	A	1	Total C O<br>4 2 2	0	0

Continued on next page...

CONFIDENTIAL VALIDATION REPORT

Continued from previous page...

Mol	Chain	Residues	Atoms	ZeroOcc	AltConf
4	A	1	Total C O<br>4 2 2	0	0
4	A	1	Total C O<br>4 2 2	0	0
4	A	1	Total C O<br>4 2 2	0	0
4	A	1	Total C O<br>4 2 2	0	0
4	A	1	Total C O<br>4 2 2	0	0
4	A	1	Total C O<br>4 2 2	0	0
4	A	1	Total C O<br>4 2 2	0	0
4	A	1	Total C O<br>4 2 2	0	0
4	B	1	Total C O<br>4 2 2	0	0
4	B	1	Total C O<br>4 2 2	0	0

- Molecule 5 is CHLORIDE ION (three-letter code: CL) (formula: Cl).

Mol	Chain	Residues	Atoms	ZeroOcc	AltConf
5	B	1	Total Cl<br>1 1	0	0
5	A	1	Total Cl<br>1 1	0	0

- Molecule 6 is BETA-D-GLUCOSE (three-letter code: BGC) (formula: $C_6H_{12}O_6$).

CONFIDENTIAL VALIDATION REPORT

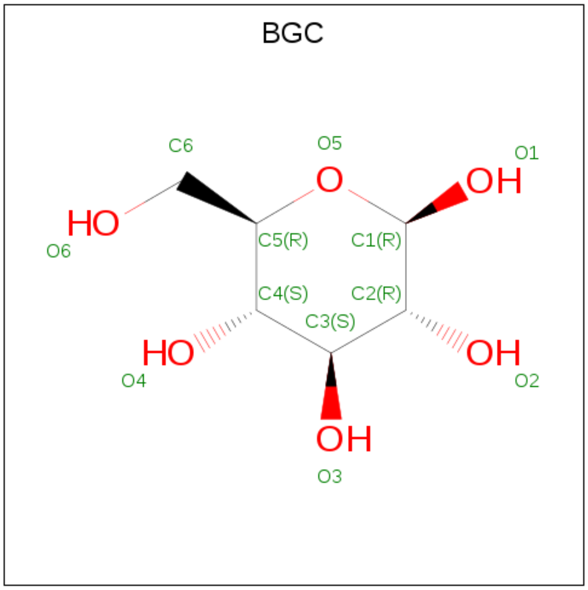

Mol	Chain	Residues	Atoms	ZeroOcc	AltConf
6	A	1	Total C O<br>12 6 6	0	0
6	A	1	Total C O<br>11 6 5	0	0
6	A	1	Total C O<br>11 6 5	0	0
6	A	1	Total C O<br>11 6 5	0	0
6	A	1	Total C O<br>11 6 5	0	0
6	A	1	Total C O<br>11 6 5	0	0
6	B	1	Total C O<br>12 6 6	0	0
6	B	1	Total C O<br>11 6 5	0	0
6	B	1	Total C O<br>11 6 5	0	0
6	B	1	Total C O<br>11 6 5	0	0
6	B	1	Total C O<br>11 6 5	0	0
6	B	1	Total C O<br>11 6 5	0	0

- Molecule 7 is water.

CONFIDENTIAL VALIDATION REPORT

Mol	Chain	Residues	Atoms	ZeroOcc	AltConf
7	A	493	Total O<br>517 517	0	24
7	B	206	Total O<br>213 213	0	7

CONFIDENTIAL VALIDATION REPORT

3 Residue-property plots

These plots are drawn for all protein, RNA and DNA chains in the entry. The first graphic for a chain summarises the proportions of the various outlier classes displayed in the second graphic. The second graphic shows the sequence view annotated by issues in geometryand electron density. Residues are color-coded according to the number of geometric quality criteria for which they contain at least one outlier: green = 0, yellow = 1, orange = 2 and red = 3 or more. A red dot above a residue indicates a poor fit to the electron density (RSRZ > 2). Stretches of 2 or more consecutive residues without any outlier are shown as a green connector. Residues present in the sample, but not in the model, are shown in grey.

- Molecule 1: Beta-1,3-oligosaccharide phosphorylase

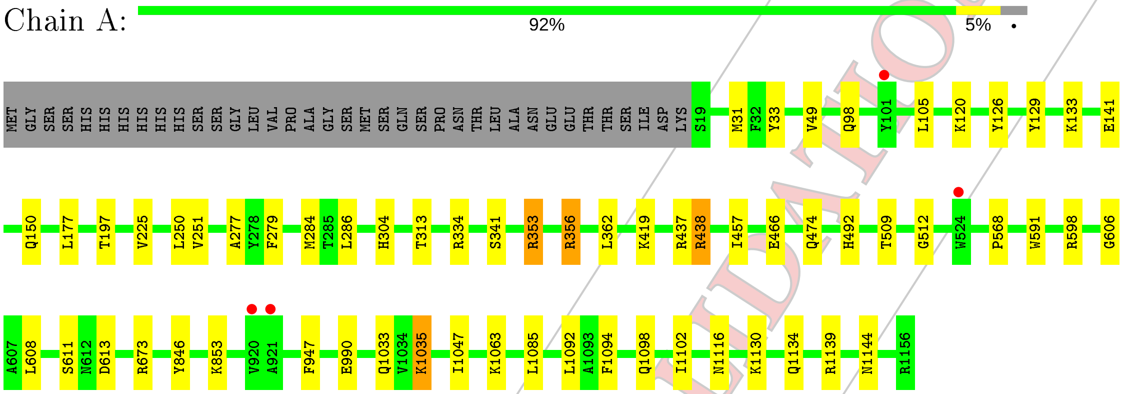

- Molecule 1: Beta-1,3-oligosaccharide phosphorylase

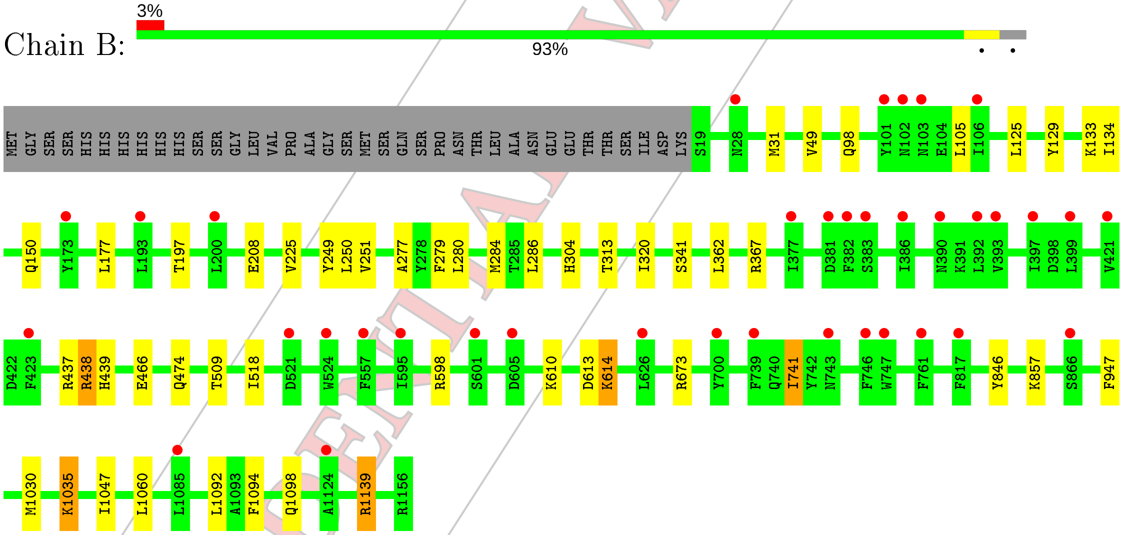

CONFIDENTIAL VALIDATION REPORT

4 Data and refinement statistics (i)

Property	Value	Source
Space group	P 21 21 21	Depositor
Cell constants<br>a, b, c, α, β, γ	99.13Å 158.76Å 178.89Å<br>90.00° 90.00° 90.00°	Depositor
Resolution (Å)	66.41 – 2.25<br>66.41 – 2.25	Depositor<br>EDS
% Data completeness<br>(in resolution range)	100.0 (66.41-2.25)<br>100.0 (66.41-2.25)	Depositor<br>EDS
R_{merge}	0.13	Depositor
R_{sym}	(Not available)	Depositor
$< I/\sigma(I) >$ [1]	1.73 (at 2.25Å)	Xtriage
Refinement program	REFMAC	Depositor
R, R_{free}	0.180 , 0.217<br>0.184 , 0.219	Depositor<br>DCC
R_{free} test set	6752 reflections (5.03%)	wwPDB-VP
Wilson B-factor (Å^2)	46.6	Xtriage
Anisotropy	0.385	Xtriage
Bulk solvent k_{sol}(e/Å^3), B_{sol}(Å^2)	0.32 , 47.1	EDS
L-test for twinning[2]	$< \|L\| > = 0.50$, $< L^2 > = 0.33$	Xtriage
Estimated twinning fraction	No twinning to report.	Xtriage
F_o,F_c correlation	0.96	EDS
Total number of atoms	18945	wwPDB-VP
Average B, all atoms (Å^2)	59.0	wwPDB-VP

Xtriage's analysis on translational NCS is as follows: *The largest off-origin peak in the Patterson function is 2.20% of the height of the origin peak. No significant pseudotranslation is detected.*

[1]Intensities estimated from amplitudes.

[2]Theoretical values of $< |L| >$, $< L^2 >$ for acentric reflections are 0.5, 0.333 respectively for untwinned datasets, and 0.375, 0.2 for perfectly twinned datasets.

CONFIDENTIAL VALIDATION REPORT

5 Model quality (i)

5.1 Standard geometry (i)

Bond lengths and bond angles in the following residue types are not validated in this section: SO4, BCN, BGC, EDO, CL

The Z score for a bond length (or angle) is the number of standard deviations the observed value is removed from the expected value. A bond length (or angle) with $|Z| > 5$ is considered an outlier worth inspection. RMSZ is the root-mean-square of all Z scores of the bond lengths (or angles).

Mol	Chain	Bond lengths		Bond angles	
		RMSZ	#$\|Z\|$ >5	RMSZ	#$\|Z\|$ >5
1	A	0.54	0/9270	0.66	3/12549 (0.0%)
1	B	0.46	0/9129	0.62	1/12391 (0.0%)
All	All	0.50	0/18399	0.64	4/24940 (0.0%)

Chiral center outliers are detected by calculating the chiral volume of a chiral center and verifying if the center is modelled as a planar moiety or with the opposite hand.A planarity outlier is detected by checking planarity of atoms in a peptide group, atoms in a mainchain group or atoms of a sidechain that are expected to be planar.

Mol	Chain	#Chirality outliers	#Planarity outliers
1	A	0	3
1	B	0	2
All	All	0	5

There are no bond length outliers.

All (4) bond angle outliers are listed below:

Mol	Chain	Res	Type	Atoms	Z	Observed(°)	Ideal(°)
1	A	334	ARG	NE-CZ-NH1	5.90	123.25	120.30
1	A	1139	ARG	NE-CZ-NH2	-5.87	117.36	120.30
1	B	1139	ARG	NE-CZ-NH2	-5.71	117.44	120.30
1	A	356	ARG	NE-CZ-NH2	-5.01	117.80	120.30

There are no chirality outliers.

All (5) planarity outliers are listed below:

Mol	Chain	Res	Type	Group
1	A	353	ARG	Sidechain
1	A	438	ARG	Sidechain

Continued on next page...

CONFIDENTIAL VALIDATION REPORT

Continued from previous page...

Mol	Chain	Res	Type	Group
1	A	673	ARG	Sidechain
1	B	438	ARG	Sidechain
1	B	673	ARG	Sidechain

5.2 Too-close contacts (i)

In the following table, the Non-H and H(model) columns list the number of non-hydrogen atoms and hydrogen atoms in the chain respectively. The H(added) column lists the number of hydrogen atoms added and optimized by MolProbity. The Clashes column lists the number of clashes within the asymmetric unit, whereas Symm-Clashes lists symmetry related clashes.

Mol	Chain	Non-H	H(model)	H(added)	Clashes	Symm-Clashes
1	A	9067	0	8721	32	0
1	B	8926	0	8422	28	0
2	A	10	0	0	0	0
2	B	10	0	0	0	0
3	A	11	0	12	0	0
3	B	11	0	12	0	0
4	A	36	0	54	2	0
4	B	8	0	12	0	0
5	A	1	0	0	0	0
5	B	1	0	0	0	0
6	A	67	0	57	1	0
6	B	67	0	57	0	0
7	A	517	0	0	3	0
7	B	213	0	0	1	0
All	All	18945	0	17347	59	0

The all-atom clashscore is defined as the number of clashes found per 1000 atoms (including hydrogen atoms). The all-atom clashscore for this structure is 2.

All (59) close contacts within the same asymmetric unit are listed below, sorted by their clash magnitude.

Atom-1	Atom-2	Interatomic distance (Å)	Clash overlap (Å)
1:A:304[A]:HIS:ND1	7:A:1301:HOH:O	1.89	1.06
1:A:356:ARG:HH22	4:A:1206:EDO:H21	1.55	0.72
1:A:120:LYS:HG3	1:A:141:GLU:HG3	1.74	0.70
1:B:304[A]:HIS:ND1	7:B:1301:HOH:O	2.32	0.60
1:A:31:MET:HB3	1:A:129:TYR:CZ	2.39	0.58
1:B:613:ASP:O	1:B:614:LYS:CB	2.51	0.58

Continued on next page...

CONFIDENTIAL VALIDATION REPORT

Continued from previous page...

Atom-1	Atom-2	Interatomic distance (Å)	Clash overlap (Å)
1:B:31:MET:HB3	1:B:129:TYR:CZ	2.40	0.56
1:B:367:ARG:HA	1:B:1030:MET:HE3	1.87	0.55
1:A:133:LYS:HD3	1:A:150:GLN:NE2	2.22	0.54
1:A:466:GLU:HB2	1:A:509:THR:HG22	1.88	0.54
1:A:853:LYS:HB3	1:B:208:GLU:OE1	2.09	0.53
1:B:439:HIS:HB3	1:B:1030:MET:CE	2.39	0.52
1:A:598:ARG:NH1	1:A:608:LEU:O	2.40	0.52
1:B:439:HIS:HB3	1:B:1030:MET:HE1	1.91	0.52
1:B:98:GLN:HB3	1:B:105:LEU:HD11	1.93	0.51
1:A:49:VAL:HG21	1:A:362:LEU:HD21	1.94	0.50
1:B:313:THR:CG2	1:B:320:ILE:HD13	2.43	0.49
1:B:598:ARG:HH22	1:B:610:LYS:CB	2.25	0.49
1:B:1092:LEU:C	1:B:1092:LEU:HD12	2.34	0.48
1:B:49:VAL:HG21	1:B:362:LEU:HD21	1.96	0.47
1:A:492:HIS:HD2	7:A:1443:HOH:O	1.98	0.47
1:A:1092:LEU:C	1:A:1092:LEU:HD12	2.34	0.47
1:A:474:GLN:O	1:A:1035:LYS:HG3	2.15	0.47
1:A:1116:ASN:HA	1:A:1144:ASN:HD21	1.80	0.46
1:B:474:GLN:O	1:B:1035:LYS:HG3	2.16	0.45
1:A:284:MET:HE3	1:A:286:LEU:HD13	1.97	0.45
1:B:250:LEU:HB2	1:B:279:PHE:HB2	1.99	0.45
1:B:133:LYS:HD3	1:B:150:GLN:NE2	2.32	0.45
1:A:251:VAL:HA	1:A:277:ALA:O	2.17	0.45
1:A:49:VAL:HG21	1:A:362:LEU:CD2	2.46	0.44
1:A:598:ARG:NH1	1:A:606:GLY:O	2.50	0.44
1:B:1047:ILE:HD11	1:B:1094:PHE:CE1	2.53	0.44
1:B:284:MET:CE	1:B:286:LEU:HD13	2.48	0.44
1:B:466:GLU:HB2	1:B:509:THR:HG22	2.00	0.44
1:A:250:LEU:HB2	1:A:279:PHE:HB2	1.99	0.44
1:A:133:LYS:HD3	1:A:150:GLN:HE21	1.83	0.44
1:B:251:VAL:HA	1:B:277:ALA:O	2.18	0.44
1:B:249:TYR:CD1	1:B:280:LEU:HD23	2.53	0.44
1:A:284:MET:CE	1:A:286:LEU:HD13	2.47	0.44
1:B:313:THR:HG21	1:B:320:ILE:HD13	2.00	0.43
1:B:741:ILE:N	1:B:741:ILE:HD13	2.32	0.43
1:B:177:LEU:C	1:B:177:LEU:HD12	2.39	0.43
1:A:1085:LEU:HD13	1:A:1102:ILE:HD11	2.00	0.43
1:A:98:GLN:HB3	1:A:105:LEU:HD11	1.99	0.43
1:A:1130:LYS:O	1:A:1134:GLN:HG2	2.18	0.43
1:A:1047:ILE:HD11	1:A:1094:PHE:CE1	2.54	0.42
1:B:1098:GLN:OE1	1:B:1139:ARG:NH2	2.52	0.42

Continued on next page...

CONFIDENTIAL VALIDATION REPORT

Continued from previous page...

Atom-1	Atom-2	Interatomic distance (Å)	Clash overlap (Å)
1:B:125:LEU:HD11	1:B:134:ILE:HD11	2.02	0.42
1:A:611:SER:OG	1:A:613:ASP:OD1	2.31	0.42
1:A:990:GLU:HG3	6:A:1215:BGC:H2	2.02	0.41
1:A:177:LEU:HD12	1:A:177:LEU:C	2.41	0.41
1:B:49:VAL:HG21	1:B:362:LEU:CD2	2.50	0.41
1:A:568:PRO:HA	1:A:591:TRP:CE3	2.55	0.41
1:B:284:MET:HE3	1:B:286:LEU:HD13	2.02	0.41
1:A:341:SER:HA	1:A:1098:GLN:OE1	2.21	0.41
1:A:1033:GLN:OE1	4:A:1206:EDO:C2	2.69	0.41
1:A:304[A]:HIS:CE1	7:A:1301:HOH:O	2.55	0.41
1:A:33:TYR:O	1:A:126:TYR:HA	2.22	0.40
1:B:341:SER:HA	1:B:1098:GLN:OE1	2.21	0.40

There are no symmetry-related clashes.

5.3 Torsion angles (i)

5.3.1 Protein backbone (i)

In the following table, the Percentiles column shows the percent Ramachandran outliers of the chain as a percentile score with respect to all X-ray entries followed by that with respect to entries of similar resolution.

The Analysed column shows the number of residues for which the backbone conformation was analysed, and the total number of residues.

Mol	Chain	Analysed	Favoured	Allowed	Outliers	Percentiles	
1	A	1137/1175 (97%)	1095 (96%)	40 (4%)	2 (0%)	49	57
1	B	1137/1175 (97%)	1095 (96%)	41 (4%)	1 (0%)	53	62
All	All	2274/2350 (97%)	2190 (96%)	81 (4%)	3 (0%)	53	62

All (3) Ramachandran outliers are listed below:

Mol	Chain	Res	Type
1	B	614	LYS
1	A	457	ILE
1	A	512	GLY

CONFIDENTIAL VALIDATION REPORT

5.3.2 Protein sidechains ⓘ

In the following table, the Percentiles column shows the percent sidechain outliers of the chain as a percentile score with respect to all X-ray entries followed by that with respect to entries of similar resolution.

The Analysed column shows the number of residues for which the sidechain conformation was analysed, and the total number of residues.

Mol	Chain	Analysed	Rotameric	Outliers	Percentiles	
1	A	974/1041 (94%)	963 (99%)	11 (1%)	76	84
1	B	939/1041 (90%)	928 (99%)	11 (1%)	74	82
All	All	1913/2082 (92%)	1891 (99%)	22 (1%)	74	82

All (22) residues with a non-rotameric sidechain are listed below:

Mol	Chain	Res	Type
1	A	197	THR
1	A	225	VAL
1	A	313	THR
1	A	353	ARG
1	A	419	LYS
1	A	437	ARG
1	A	438	ARG
1	A	846	TYR
1	A	947	PHE
1	A	1035	LYS
1	A	1063	LYS
1	B	197	THR
1	B	225	VAL
1	B	437	ARG
1	B	438	ARG
1	B	518	ILE
1	B	741	ILE
1	B	846	TYR
1	B	857	LYS
1	B	947	PHE
1	B	1035	LYS
1	B	1060	LEU

Some sidechains can be flipped to improve hydrogen bonding and reduce clashes. All (19) such sidechains are listed below:

CONFIDENTIAL VALIDATION REPORT

Mol	Chain	Res	Type
1	A	100	GLN
1	A	150	GLN
1	A	156	GLN
1	A	194	GLN
1	A	684	ASN
1	A	689	ASN
1	A	740	GLN
1	A	965	GLN
1	A	996	ASN
1	A	1051	ASN
1	A	1144	ASN
1	B	100	GLN
1	B	156	GLN
1	B	194	GLN
1	B	684	ASN
1	B	689	ASN
1	B	743	ASN
1	B	965	GLN
1	B	996	ASN

5.3.3 RNA (i)

There are no RNA molecules in this entry.

5.4 Non-standard residues in protein, DNA, RNA chains (i)

There are no non-standard protein/DNA/RNA residues in this entry.

5.5 Carbohydrates (i)

There are no carbohydrates in this entry.

5.6 Ligand geometry (i)

Of 31 ligands modelled in this entry, 2 are monoatomic - leaving 29 for Mogul analysis.

In the following table, the Counts columns list the number of bonds (or angles) for which Mogul statistics could be retrieved, the number of bonds (or angles) that are observed in the model and the number of bonds (or angles) that are defined in the Chemical Component Dictionary. The Link column lists molecule types, if any, to which the group is linked. The Z score for a bond length (or angle) is the number of standard deviations the observed value is removed from the

CONFIDENTIAL VALIDATION REPORT

expected value. A bond length (or angle) with $|Z| > 2$ is considered an outlier worth inspection. RMSZ is the root-mean-square of all Z scores of the bond lengths (or angles).

Mol	Type	Chain	Res	Link	Bond lengths			Bond angles		
					Counts	RMSZ	#|Z| > 2	Counts	RMSZ	#|Z| > 2
2	SO4	A	1201	-	4,4,4	0.48	0	6,6,6	0.44	0
2	SO4	A	1202	-	4,4,4	0.21	0	6,6,6	0.58	0
3	BCN	A	1203	-	7,10,10	0.37	0	8,11,11	1.24	1 (12%)
4	EDO	A	1204	-	3,3,3	0.37	0	2,2,2	0.48	0
4	EDO	A	1205	-	3,3,3	0.50	0	2,2,2	0.37	0
4	EDO	A	1206	-	3,3,3	0.26	0	2,2,2	0.62	0
4	EDO	A	1207	-	3,3,3	0.44	0	2,2,2	0.27	0
4	EDO	A	1208	-	3,3,3	0.38	0	2,2,2	0.56	0
4	EDO	A	1209	-	3,3,3	0.49	0	2,2,2	0.35	0
4	EDO	A	1210	-	3,3,3	0.47	0	2,2,2	0.57	0
4	EDO	A	1211	-	3,3,3	0.53	0	2,2,2	0.09	0
4	EDO	A	1212	-	3,3,3	0.39	0	2,2,2	0.22	0
6	BGC	A	1214	6	12,12,12	0.31	0	17,17,17	0.68	0
6	BGC	A	1215	6	11,11,12	0.51	0	15,15,17	1.01	1 (6%)
6	BGC	A	1216	6	11,11,12	0.39	0	15,15,17	0.65	0
6	BGC	A	1217	6	11,11,12	0.43	0	15,15,17	0.62	0
6	BGC	A	1218	6	11,11,12	0.44	0	15,15,17	0.68	0
6	BGC	A	1219	6	11,11,12	0.46	0	15,15,17	0.49	0
2	SO4	B	1201	-	4,4,4	0.49	0	6,6,6	0.41	0
2	SO4	B	1202	-	4,4,4	0.44	0	6,6,6	0.22	0
3	BCN	B	1203	-	7,10,10	0.36	0	8,11,11	0.94	1 (12%)
4	EDO	B	1204	-	3,3,3	0.50	0	2,2,2	0.09	0
4	EDO	B	1205	-	3,3,3	0.51	0	2,2,2	0.27	0
6	BGC	B	1207	6	12,12,12	0.41	0	17,17,17	0.60	0
6	BGC	B	1208	6	11,11,12	0.45	0	15,15,17	0.84	1 (6%)
6	BGC	B	1209	6	11,11,12	0.28	0	15,15,17	0.50	0
6	BGC	B	1210	6	11,11,12	0.42	0	15,15,17	0.51	0
6	BGC	B	1211	6	11,11,12	0.37	0	15,15,17	0.62	0
6	BGC	B	1212	6	11,11,12	0.38	0	15,15,17	0.60	0

In the following table, the Chirals column lists the number of chiral outliers, the number of chiral centers analysed, the number of these observed in the model and the number defined in the Chemical Component Dictionary. Similar counts are reported in the Torsion and Rings columns. '-' means no outliers of that kind were identified.

Mol	Type	Chain	Res	Link	Chirals	Torsions	Rings
2	SO4	A	1201	-	-	0/0/0/0	0/0/0/0
2	SO4	A	1202	-	-	0/0/0/0	0/0/0/0
3	BCN	A	1203	-	-	0/8/10/10	0/0/0/0

Continued on next page...

CONFIDENTIAL VALIDATION REPORT

Continued from previous page...

Mol	Type	Chain	Res	Link	Chirals	Torsions	Rings
4	EDO	A	1204	-	-	0/1/1/1	0/0/0/0
4	EDO	A	1205	-	-	0/1/1/1	0/0/0/0
4	EDO	A	1206	-	-	0/1/1/1	0/0/0/0
4	EDO	A	1207	-	-	0/1/1/1	0/0/0/0
4	EDO	A	1208	-	-	0/1/1/1	0/0/0/0
4	EDO	A	1209	-	-	0/1/1/1	0/0/0/0
4	EDO	A	1210	-	-	0/1/1/1	0/0/0/0
4	EDO	A	1211	-	-	0/1/1/1	0/0/0/0
4	EDO	A	1212	-	-	0/1/1/1	0/0/0/0
6	BGC	A	1214	6	-	0/2/22/22	0/1/1/1
6	BGC	A	1215	6	-	0/2/19/22	0/1/1/1
6	BGC	A	1216	6	-	0/2/19/22	0/1/1/1
6	BGC	A	1217	6	-	0/2/19/22	0/1/1/1
6	BGC	A	1218	6	-	0/2/19/22	0/1/1/1
6	BGC	A	1219	6	-	0/2/19/22	0/1/1/1
2	SO4	B	1201	-	-	0/0/0/0	0/0/0/0
2	SO4	B	1202	-	-	0/0/0/0	0/0/0/0
3	BCN	B	1203	-	-	0/8/10/10	0/0/0/0
4	EDO	B	1204	-	-	0/1/1/1	0/0/0/0
4	EDO	B	1205	-	-	0/1/1/1	0/0/0/0
6	BGC	B	1207	6	-	0/2/22/22	0/1/1/1
6	BGC	B	1208	6	-	0/2/19/22	0/1/1/1
6	BGC	B	1209	6	-	0/2/19/22	0/1/1/1
6	BGC	B	1210	6	-	0/2/19/22	0/1/1/1
6	BGC	B	1211	6	-	0/2/19/22	0/1/1/1
6	BGC	B	1212	6	-	0/2/19/22	0/1/1/1

There are no bond length outliers.

All (4) bond angle outliers are listed below:

Mol	Chain	Res	Type	Atoms	Z	Observed(^{o})	Ideal(^{o})
3	A	1203	BCN	C2-C1-N1	-2.92	109.32	113.48
3	B	1203	BCN	C2-C1-N1	-2.46	109.98	113.48
6	B	1208	BGC	C1-C2-C3	2.27	112.53	109.66
6	A	1215	BGC	C1-C2-C3	2.43	112.74	109.66

There are no chirality outliers.

There are no torsion outliers.

There are no ring outliers.

2 monomers are involved in 3 short contacts:

CONFIDENTIAL VALIDATION REPORT

Mol	Chain	Res	Type	Clashes	Symm-Clashes
4	A	1206	EDO	2	0
6	A	1215	BGC	1	0

5.7 Other polymers (i)

There are no such residues in this entry.

5.8 Polymer linkage issues (i)

There are no chain breaks in this entry.

CONFIDENTIAL VALIDATION REPORT

6 Fit of model and data (i)

6.1 Protein, DNA and RNA chains (i)

In the following table, the column labelled '#RSRZ> 2' contains the number (and percentage) of RSRZ outliers, followed by percent RSRZ outliers for the chain as percentile scores relative to all X-ray entries and entries of similar resolution. The OWAB column contains the minimum, median, 95th percentile and maximum values of the occupancy-weighted average B-factor per residue. The column labelled 'Q< 0.9' lists the number of (and percentage) of residues with an average occupancy less than 0.9.

Mol	Chain	Analysed	<RSRZ>	#RSRZ>2			OWAB(Å^2)	Q<0.9
1	A	1138/1175 (96%)	-0.25	4 (0%)	92	93	33, 46, 82, 130	0
1	B	1138/1175 (96%)	0.05	37 (3%)	46	49	41, 68, 105, 150	0
All	All	2276/2350 (96%)	-0.10	41 (1%)	68	72	33, 56, 99, 150	0

All (41) RSRZ outliers are listed below:

Mol	Chain	Res	Type	RSRZ
1	B	101	TYR	5.6
1	A	101	TYR	4.4
1	B	386	ILE	3.8
1	A	920	VAL	3.7
1	A	524	TRP	3.7
1	B	524	TRP	3.6
1	B	102	ASN	3.5
1	B	1124	ALA	3.5
1	B	746	PHE	3.4
1	B	377	ILE	3.4
1	B	382	PHE	3.2
1	B	106	ILE	3.1
1	B	626	LEU	2.8
1	B	595	ILE	2.8
1	B	739	PHE	2.8
1	B	393	VAL	2.8
1	B	521	ASP	2.6
1	B	390	ASN	2.6
1	B	392	LEU	2.6
1	B	423	PHE	2.6
1	B	557	PHE	2.6
1	B	28	ASN	2.5
1	B	397	ILE	2.5
1	B	747	TRP	2.3

Continued on next page...

CONFIDENTIAL VALIDATION REPORT

Continued from previous page...

Mol	Chain	Res	Type	RSRZ
1	B	399	LEU	2.3
1	B	601	SER	2.3
1	B	761	PHE	2.2
1	B	743	ASN	2.2
1	B	605	ASP	2.2
1	B	866	SER	2.1
1	B	700	TYR	2.1
1	B	103	ASN	2.1
1	B	383	SER	2.1
1	A	921	ALA	2.1
1	B	421	VAL	2.1
1	B	173	TYR	2.1
1	B	193	LEU	2.0
1	B	381	ASP	2.0
1	B	200	LEU	2.0
1	B	1085	LEU	2.0
1	B	817	PHE	2.0

6.2 Non-standard residues in protein, DNA, RNA chains (i)

There are no non-standard protein/DNA/RNA residues in this entry.

6.3 Carbohydrates (i)

There are no carbohydrates in this entry.

6.4 Ligands (i)

In the following table, the Atoms column lists the number of modelled atoms in the group and the number defined in the chemical component dictionary. The B-factors column lists the minimum, median, 95th percentile and maximum values of B factors of atoms in the group. The column labelled 'Q< 0.9' lists the number of atoms with occupancy less than 0.9.

Mol	Type	Chain	Res	Atoms	RSCC	RSR	B-factors($Å^2$)	Q<0.9
6	BGC	B	1207	12/12	0.81	0.15	77,88,94,94	0
4	EDO	B	1205	4/4	0.87	0.15	72,75,78,78	0
5	CL	B	1206	1/1	0.88	0.36	92,92,92,92	0
2	SO4	B	1201	5/5	0.90	0.19	91,91,95,97	0
4	EDO	A	1207	4/4	0.91	0.20	57,57,60,61	0
4	EDO	A	1211	4/4	0.91	0.18	53,55,56,58	0

Continued on next page...

CONFIDENTIAL VALIDATION REPORT

Continued from previous page...

Mol	Type	Chain	Res	Atoms	RSCC	RSR	B-factors(Å^2)	Q<0.9
3	BCN	B	1203	11/11	0.92	0.18	53,66,72,75	0
4	EDO	A	1205	4/4	0.93	0.16	49,49,53,54	0
4	EDO	A	1208	4/4	0.93	0.27	52,58,65,68	0
4	EDO	A	1210	4/4	0.94	0.15	59,70,71,77	0
2	SO4	B	1202	5/5	0.94	0.13	90,93,97,106	0
6	BGC	A	1219	11/12	0.94	0.10	49,57,59,59	0
4	EDO	A	1212	4/4	0.94	0.14	60,61,64,67	0
4	EDO	B	1204	4/4	0.94	0.16	64,66,66,68	0
6	BGC	B	1208	11/12	0.94	0.08	67,72,79,80	0
6	BGC	A	1215	11/12	0.95	0.09	52,56,57,60	0
5	CL	A	1213	1/1	0.95	0.22	78,78,78,78	0
6	BGC	A	1214	12/12	0.95	0.10	60,64,66,66	0
6	BGC	B	1209	11/12	0.95	0.09	60,64,65,68	0
6	BGC	B	1210	11/12	0.95	0.09	57,66,73,74	0
6	BGC	B	1212	11/12	0.95	0.11	56,60,68,69	0
3	BCN	A	1203	11/11	0.95	0.14	40,49,55,60	0
6	BGC	A	1218	11/12	0.96	0.13	47,51,55,55	0
6	BGC	A	1217	11/12	0.97	0.10	51,59,66,68	0
4	EDO	A	1204	4/4	0.97	0.12	41,42,42,43	0
4	EDO	A	1206	4/4	0.97	0.21	52,53,54,59	0
4	EDO	A	1209	4/4	0.98	0.15	42,44,45,51	0
6	BGC	A	1216	11/12	0.98	0.08	49,52,55,56	0
6	BGC	B	1211	11/12	0.98	0.10	55,58,59,60	0
2	SO4	A	1201	5/5	0.98	0.14	59,59,64,65	0
2	SO4	A	1202	5/5	0.99	0.13	51,52,55,56	0

6.5 Other polymers ⓘ

There are no such residues in this entry.

CONFIDENTIAL VALIDATION REPORT
